# [Creatinine] can change in an unexpected direction due to the volume change rate that interacts with kinetic GFR: Potentially positive paradox

**DOI:** 10.14814/phy2.15172

**Published:** 2022-02-10

**Authors:** Sheldon Chen, Robert Chiaramonte

**Affiliations:** ^1^ Section of Nephrology MD Anderson Cancer Center Houston Texas USA; ^2^ Internal Medicine New York New York USA

**Keywords:** creatinine clearance, CRRT, derivative, differential equation

## Abstract

[Creatinine] was proved to change in the opposite direction of the kinetic GFR (GFR_K_), but does the [creatinine] also change in the opposite direction of the volume rate? If volume is administered and the [creatinine] actually goes up, then the two changes move in the same direction and their ratio is positive, paradoxically. The equation that describes [creatinine] as a function of time was differentiated with respect to the volume rate. This partial first derivative has a global maximum that can be positive under definable conditions. Knowing what makes the maximum positive informs when the derivative will be positive over some continuous domain of volume rate inputs. The first derivative versus volume rate curve has a maximum and a minimum point depending on the GFR_K_. If GFR_K_ is below a calculable value, then the curve's minimum vanishes, letting it descend to ‐∞ and not allowing the derivative to ever be positive. If GFR_K_ lies between a lower and a higher calculable value, then the curve's maximum vanishes, letting the derivative diverge to +∞, though the clinical scenario is unrealistic. If GFR_K_ is above the higher calculable value, then the curve's absolute maximum can become positive by decreasing the creatinine generation rate or increasing the initial [creatinine]. The derivative is potentially positive under these clinically realizable circumstances. The combination of parameters above can align in septic patients (low creatinine generation rate) with kidney failure (high initial [creatinine]) who are put on continuous dialysis (high GFR_K_). If a first derivative is positive, removing more volume can improve the [creatinine] and, dismayingly, giving more volume can worsen the [creatinine]. This paradox is explained by a covert interplay between the ambient [creatinine] and GFR_K_ that excretes creatinine faster than its volume of distribution declines.

## Graphical abstract

The [creatinine] can move in the same direction as the volume rate, counterintuitively, meaning that volume gain can sometimes increase the [creatinine] and vice versa. These phenomena, thankfully rare, are allowed by the math and need not invoke any confounding factors like a change in kidney function.

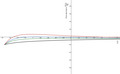

## 1 INTRODUCTION

We previously showed that changes in the glomerular filtration rate (GFR) must always drive the serum creatinine in the opposite direction (Chen & Chiaramonte, 2021). If the GFR were to decrease, then the creatinine would have to increase, and vice versa. Though these statements seem obvious, they were only recently proved by differentiating the creatinine with respect to kinetic GFR (Chen, 2013) and finding that this partial derivative's sign is always negative for any possible set of real‐world values. It does not matter how extreme the variables are or how they are combined. The perpetual negative sign assures doctors that a rise in creatinine (positive Δcreatinine) had to come about from a drop in kinetic GFR (negative ΔGFR_K_) and vice versa, with all else being constant—particularly time.

Is creatinine also related to the volume change rate in an ever‐opposing way? If more volume is being gained (positive Δvolume rate), the [creatinine] is further diluted and $\Delta \left[Cr\right]$ is negative. If more volume is being lost (negative Δvolume rate), the [creatinine] is further concentrated and $\Delta \left[Cr\right]$ is positive. With the signs being opposite in our thought experiments, it would appear that the partial derivative of $\left[Cr\right]$ with respect to volume change rate is always negative, same as for kinetic GFR (Chen & Chiaramonte, 2021). But could unsuspected factors alter the sign? What if a negative Δvolume rate concentrates the [creatinine] even further, but that enables the kinetic GFR to excrete more creatinine? Would the creatinine quantity decline faster than the volume of distribution, making the creatinine concentration fraction lower in value—a negative $\Delta \left[Cr\right]$? If so, the partial derivative would be *positive*, with volume rate and $\left[Cr\right]$ decreasing over the same time frame. The potential for a positive sign brings up a clinical paradox. Sometimes, being more aggressive with the volume removal may improve the serum creatinine (Hegde, 2020). Or, giving even more volume may increase the creatinine. These paradoxes can occur in septic patients on continuous dialysis, for purely mathematical reasons to be shown that need not involve the messiness of real life, which is more complex than the derivative model that assumes that only volume and [creatinine] can change.

### 1.1 Creatinine kinetics

The differential equation that underpins the kinetic GFR states that the rate of change in the creatinine mass is equal to the creatinine input rate minus the creatinine output rate (Chen, 2018a, 2018b; Chen & Chiaramonte, 2019). Further, the creatinine mass at any given time is the current [creatinine] times the volume of distribution, typically taken to be total body water (TBW) (Bjornsson, 1979; Chow, 1985; Edwards, 1959; Jones & Burnett, 1974; Pickering et al., 2013). To account for the concentrating and diluting effects on the [creatinine], its volume of distribution can be modeled to change at a constant rate: $V_{t}=V_{0}+\frac{\Delta V}{\Delta t}t$, where $V_{t}$ is the volume as a function of time, $V_{0}$ is the initial volume, $\frac{\Delta V}{\Delta t}$ is the (average) volume change rate, and t is time. The creatinine input rate is primarily determined by the muscle mass, which tends to be fairly stable so that the creatinine generation rate is usually thought of as a constant: Gen. The creatinine output rate is mostly determined by the kidney such that the excretion rate is equal to the kinetic GFR times the ambient [creatinine]: ${\mathrm{GFR}}_{K}\cdot {\left[Cr\right]}_{t}$, where ${\left[Cr\right]}_{t}$ is the [creatinine] at a particular time. Thus, the differential equation is:

(1) $$\frac{d}{dt}\left({\left[Cr\right]}_{t}\cdot V_{t}\right)=Gen‐{\mathrm{GFR}}_{K}\cdot {\left[Cr\right]}_{t}$$

This first‐order linear differential equation's solution, as previously published, is (Chen, 2018a):

(2) $${\left[\mathrm{Cr}\right]}_{t}={\left[\mathrm{Cr}\right]}_{0}+\underset{\text{Evolution over time}}{\underbrace{\left[1‐{\left(\frac{V_{0}}{V_{0}+\frac{\Delta V}{\Delta t}t}\right)}^{\left(1+\frac{GFR_{K}}{\frac{\Delta V}{\Delta t}}\right)}\right]}}\cdot \underset{\left[\mathrm{Cr}\right]\;\text{spread}}{\underbrace{\left(\frac{\mathrm{Gen}}{GFR_{K}+\frac{\Delta V}{\Delta t}}‐{\left[\mathrm{Cr}\right]}_{0}\right)}}$$

In other words, the serum [creatinine] at a given time is equal to the initial [creatinine] $\left({\left[Cr\right]}_{0}\right)$ plus a time‐evolved portion of the spread between the initial [creatinine] and the [creatinine] reached at a new steady state if the kinetic GFR and volume change rate remained at those levels.

## 2 MATERIALS AND METHODS

### 2.1 Derivative of [creatinine] with respect to volume change rate

From Equation (2), we can deduce how the serum creatinine would change if one other variable were tweaked, and the partial derivative is suited to this task. Previously, the one other variable was kinetic GFR (Chen & Chiaramonte, 2021), but now the one other variable will be the volume change rate. The derivative of ${\left[Cr\right]}_{t}$ with respect to $\frac{\Delta V}{\Delta t}$ quantifies their relationship at every instant, allowing a comprehensive assessment of the sign. If the sign can be positive, then ${\left[Cr\right]}_{t}$ may change in the same direction as $\frac{\Delta V}{\Delta t}$.

In Equation (2), the derivative of $\left[1‐{\left(\frac{V_{0}}{V_{0}+\frac{\Delta V}{\Delta t}t}\right)}^{\left(1+\frac{{\mathrm{GFR}}_{K}}{\frac{\Delta V}{\Delta t}}\right)}\right]$ with respect to $\frac{\Delta V}{\Delta t}$ is:

$$0‐{\left[{\left(\frac{V_{0}}{V_{0}+\frac{\Delta V}{\Delta t}t}\right)}^{\left(1+\frac{GFR_{K}}{\frac{\Delta V}{\Delta t}}\right)}\right]}^{\prime }$$

To calculate the derivative of the exponential, let $y={\left(\frac{V_{0}}{V_{0}+\frac{\Delta V}{\Delta t}t}\right)}^{\left(1+\frac{{\mathrm{GFR}}_{K}}{\frac{\Delta V}{\Delta t}}\right)}$ and use logarithms.

$$\begin{array}{c} \ln\;y=\ln{\left(\frac{V_{0}}{V_{0}+\frac{\Delta V}{\Delta t}t}\right)}^{\left(1+\frac{{\mathrm{GFR}}_{K}}{\frac{\Delta V}{\Delta t}}\right)}=\left(1+\frac{{\mathrm{GFR}}_{K}}{\frac{\Delta V}{\Delta t}}\right)\cdot \ln\left(\frac{V_{0}}{V_{0}+\frac{\Delta V}{\Delta t}t}\right) \end{array}$$

Differentiate with the product rule:

$$\begin{array}{c} \frac{1}{y}y^{\prime }=‐\frac{GFR_{K}}{{\frac{\Delta V}{\Delta t}}^{2}}\cdot \ln\left(\frac{V_{0}}{V_{0}+\frac{\Delta V}{\Delta t}t}\right)+\left(1+\frac{GFR_{K}}{\frac{\Delta V}{\Delta t}}\right)\cdot \frac{V_{0}+\frac{\Delta V}{\Delta t}t}{V_{0}}\cdot \\ \;\frac{‐V_{0}\cdot t}{{\left(V_{0}+\frac{\Delta V}{\Delta t}t\right)}^{2}} \end{array}$$

$$\frac{1}{y}y^{\prime }=‐\frac{GFR_{K}}{{\frac{\Delta V}{\Delta t}}^{2}}\cdot \ln\left(\frac{V_{0}}{V_{0}+\frac{\Delta V}{\Delta t}t}\right)‐\left(1+\frac{GFR_{K}}{\frac{\Delta V}{\Delta t}}\right)\cdot \frac{t}{V_{0}+\frac{\Delta V}{\Delta t}t}$$

$$\begin{array}{c} y^{\prime }=‐{\left(\frac{V_{0}}{V_{0}+\frac{\Delta V}{\Delta t}t}\right)}^{\left(1+\frac{GFR_{K}}{\frac{\Delta V}{\Delta t}}\right)}\cdot \\ \;\left[\frac{GFR_{K}}{{\frac{\Delta V}{\Delta t}}^{2}}\cdot \ln\left(\frac{V_{0}}{V_{0}+\frac{\Delta V}{\Delta t}t}\right)+\left(1+\frac{GFR_{K}}{\frac{\Delta V}{\Delta t}}\right)\cdot \frac{t}{V_{0}+\frac{\Delta V}{\Delta t}t}\right] \end{array}$$

(3) $$\begin{array}{c} \text{Thus},{\left[1‐{\left(\frac{V_{0}}{V_{0}+\frac{\Delta V}{\Delta t}t}\right)}^{\left(1+\frac{GFR_{K}}{\frac{\Delta V}{\Delta t}}\right)}\right]}^{\prime }={\left(\frac{V_{0}}{V_{0}+\frac{\Delta V}{\Delta t}t}\right)}^{\left(1+\frac{GFR_{K}}{\frac{\Delta V}{\Delta t}}\right)}\cdot \\ \left[\frac{GFR_{K}}{{\frac{\Delta V}{\Delta t}}^{2}}\cdot \ln\left(\frac{V_{0}}{V_{0}+\frac{\Delta V}{\Delta t}t}\right)+\left(1+\frac{GFR_{K}}{\frac{\Delta V}{\Delta t}}\right)\cdot \frac{t}{V_{0}+\frac{\Delta V}{\Delta t}t}\right] \end{array}$$

Next, in Equation (2), find the derivative of $\left(\frac{\mathrm{Gen}}{{\mathrm{GFR}}_{K}+\frac{\Delta V}{\Delta t}}‐{\left[Cr\right]}_{0}\right)$ with respect to $\frac{\Delta V}{\Delta t}$:

(4) $${\left(\frac{\mathrm{Gen}}{GFR_{K}+\frac{\Delta V}{\Delta t}}‐{\left[\mathrm{Cr}\right]}_{0}\right)}^{\prime }=‐\frac{\mathrm{Gen}}{{\left(GFR_{K}+\frac{\Delta V}{\Delta t}\right)}^{2}}$$

Putting Equations (3) and (4) together in using the product rule on Equation (2), we find that $\frac{\partial {\left[Cr\right]}_{t}}{\partial \frac{\Delta V}{\Delta t}}$ is:

(5) $$\begin{array}{c} \frac{\partial {\left[Cr\right]}_{t}}{\partial \frac{\Delta V}{\Delta t}}={\left(\frac{V_{0}}{V_{0}+\frac{\Delta V}{\Delta t}t}\right)}^{\left(1+\frac{{\mathrm{GFR}}_{K}}{\frac{\Delta V}{\Delta t}}\right)}\cdot \left[\frac{{\mathrm{GFR}}_{K}}{{\frac{\Delta V}{\Delta t}}^{2}}\cdot \ln\left(\frac{V_{0}}{V_{0}+\frac{\Delta V}{\Delta t}t}\right)+\left(1+\frac{{\mathrm{GFR}}_{K}}{\frac{\Delta V}{\Delta t}}\right)\cdot \frac{t}{V_{0}+\frac{\Delta V}{\Delta t}t}\right]\cdot \left(\frac{\mathrm{Gen}}{{\mathrm{GFR}}_{K}+\frac{\Delta V}{\Delta t}}‐{\left[Cr\right]}_{0}\right)\\ \;+\left[1‐{\left(\frac{V_{0}}{V_{0}+\frac{\Delta V}{\Delta t}t}\right)}^{\left(1+\frac{{\mathrm{GFR}}_{K}}{\frac{\Delta V}{\Delta t}}\right)}\right]\cdot \frac{‐Gen}{{\left({\mathrm{GFR}}_{K}+\frac{\Delta V}{\Delta t}\right)}^{2}} \end{array}$$

Note: Unit conversions are not shown, but the final units could be $\frac{\text{mg}}{\text{dl}}\;\text{per}\;\frac{L}{h}$, for example. The main conversion factors are $\frac{50}{3}$ and $\frac{3}{50}$. The $\frac{50}{3}$ converts $\frac{\Delta V}{\Delta t}$ in L/h to ml/min: $\frac{1000\;\text{ml}}{L}\cdot \frac{h}{60\;\text{min}}=\frac{50}{3}$, and the $\frac{3}{50}$ converts ${\mathrm{GFR}}_{K}$ in ml/min to L/h: $\frac{L}{1000\;\text{ml}}\cdot \frac{60\;\text{min}}{h}=\frac{3}{50}$.

### 2.2 Calculator and concept map

To follow the calculations in the Results, please download a spreadsheet we created to calculate the main equations in the manuscript. You can use the spreadsheet to explore your own scenarios and questions. For a map of the concepts being presented, the final algorithm in Section 3.8 may help with understanding when the first derivative in Equation (5) can be positive. First, Gen and ${\left[Cr\right]}_{0}$ will be varied (Section 3.5), as their ratio is a principal determinant of positivity. Later, ${\mathrm{GFR}}_{K}$ will also be varied (Section 3.6), as values above a calculable reference point can allow the first derivative to be positive in situations that are clinically encountered.

## 3 RESULTS

### 3.1 First derivative behavior and sign

**FIGURE 1:**
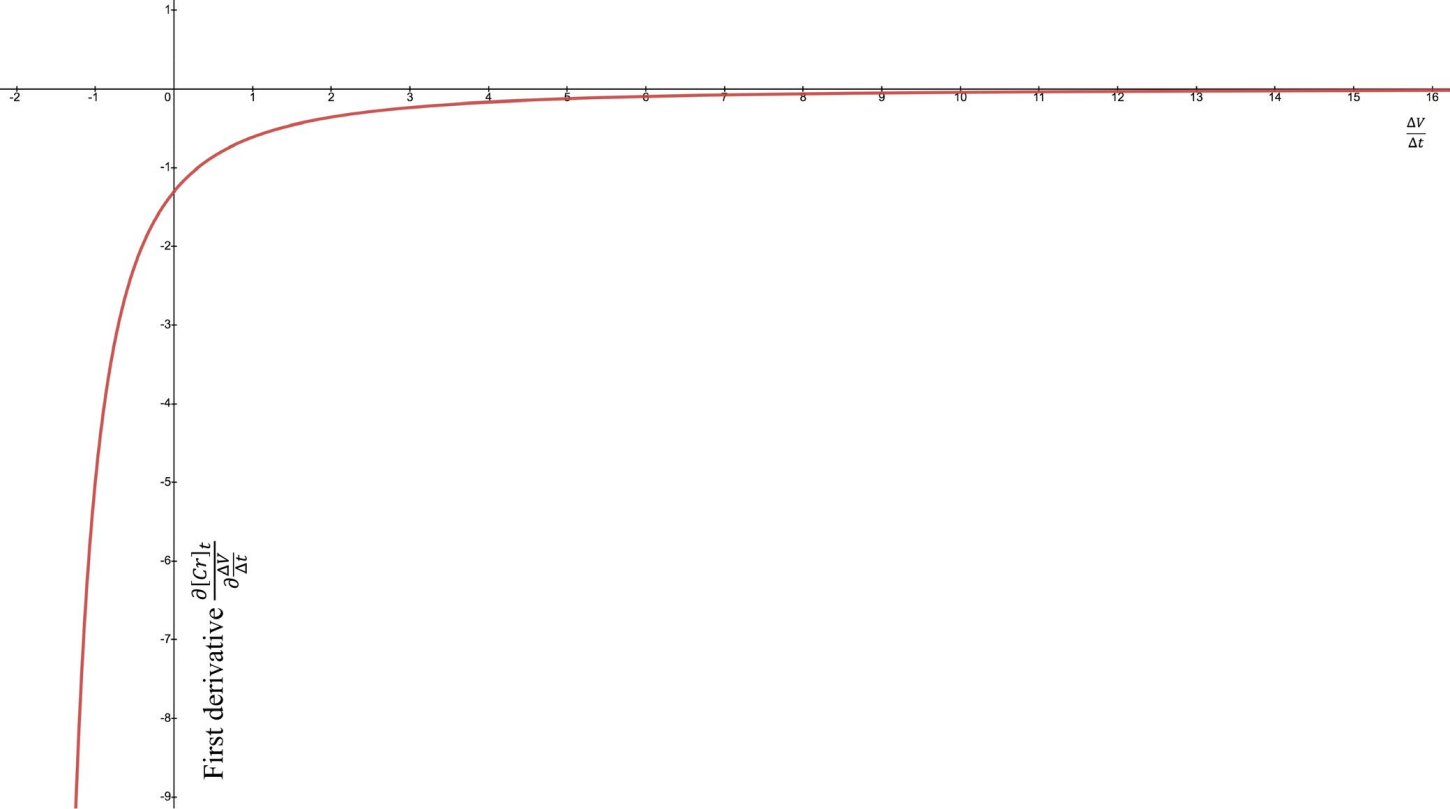
Example first derivative of [creatinine] with respect to volume change rate. Equation (5) is graphed with $\frac{\Delta V}{\Delta t}$ as an independent variable (x‐axis) and $\frac{\partial {\left[Cr\right]}_{t}}{\partial \frac{\Delta V}{\Delta t}}$ as the dependent variable (y‐axis). The other variables are Gen=100 mg/dL·mL/min, $V_{0}=42$ L, t=24 h, ${\mathrm{GFR}}_{K}=20$ mL/min, and ${\left[Cr\right]}_{0}=1$ mg/dL. From left to right, the $\frac{\partial {\left[Cr\right]}_{t}}{\partial \frac{\Delta V}{\Delta t}}$ starts at ‐∞ where $\frac{\Delta V}{\Delta t}$ approaches its leftmost value of $‐\frac{V_{0}}{t}$. The curve rises quickly but remains negative. As $\frac{\Delta V}{\Delta t}$ increases, the $\frac{\partial {\left[Cr\right]}_{t}}{\partial \frac{\Delta V}{\Delta t}}$ flattens out as it approaches zero asymptotically. The first derivative is always negative in this example of acute kidney injury

To gauge the behavior and sign of the partial first derivative, we graphed $\frac{\partial {\left[Cr\right]}_{t}}{\partial \frac{\Delta V}{\Delta t}}$ (y‐axis) vs. $\frac{\Delta V}{\Delta t}$ (x‐axis) for an acute kidney injury (AKI): steady state GFR of 100 ml/min corresponding to an initial $\left[Cr\right]$ of 1.0 mg/dl that increases over the next 24 hours when the ${\mathrm{GFR}}_{K}$ suddenly drops to 20 ml/min in a patient with a TBW (volume of distribution) of 42 L. According to the thought experiments, the sign of $\frac{\partial {\left[Cr\right]}_{t}}{\partial \frac{\Delta V}{\Delta t}}$ should be negative (below y=0) throughout the gamut of $\frac{\Delta V}{\Delta t}$ values (Figure 1). As $\frac{\Delta V}{\Delta t}$ approaches an extreme that would deplete all of the TBW by the 24‐h mark (in this case), the $\frac{\partial {\left[Cr\right]}_{t}}{\partial \frac{\Delta V}{\Delta t}}$ goes to ‐∞ (Figure 1). A volume of zero is nonsensical, so if $V_{0}+\frac{\Delta V}{\Delta t}t$ needs to be >0, then $\frac{\Delta V}{\Delta t}$ must be $>‐\frac{V_{0}}{t}$. At the far right of the graph, as $\frac{\Delta V}{\Delta t}\to +\infty$, the value of $\frac{\partial {\left[Cr\right]}_{t}}{\partial \frac{\Delta V}{\Delta t}}$ approaches zero from below, that is, $\lim_{\frac{\Delta V}{\Delta t}\to \infty }\frac{\partial {\left[Cr\right]}_{t}}{\partial \frac{\Delta V}{\Delta t}}=0$ (Figure 1). So far, $\frac{\partial {\left[Cr\right]}_{t}}{\partial \frac{\Delta V}{\Delta t}}$ seems to always be negative.

**FIGURE 2:**
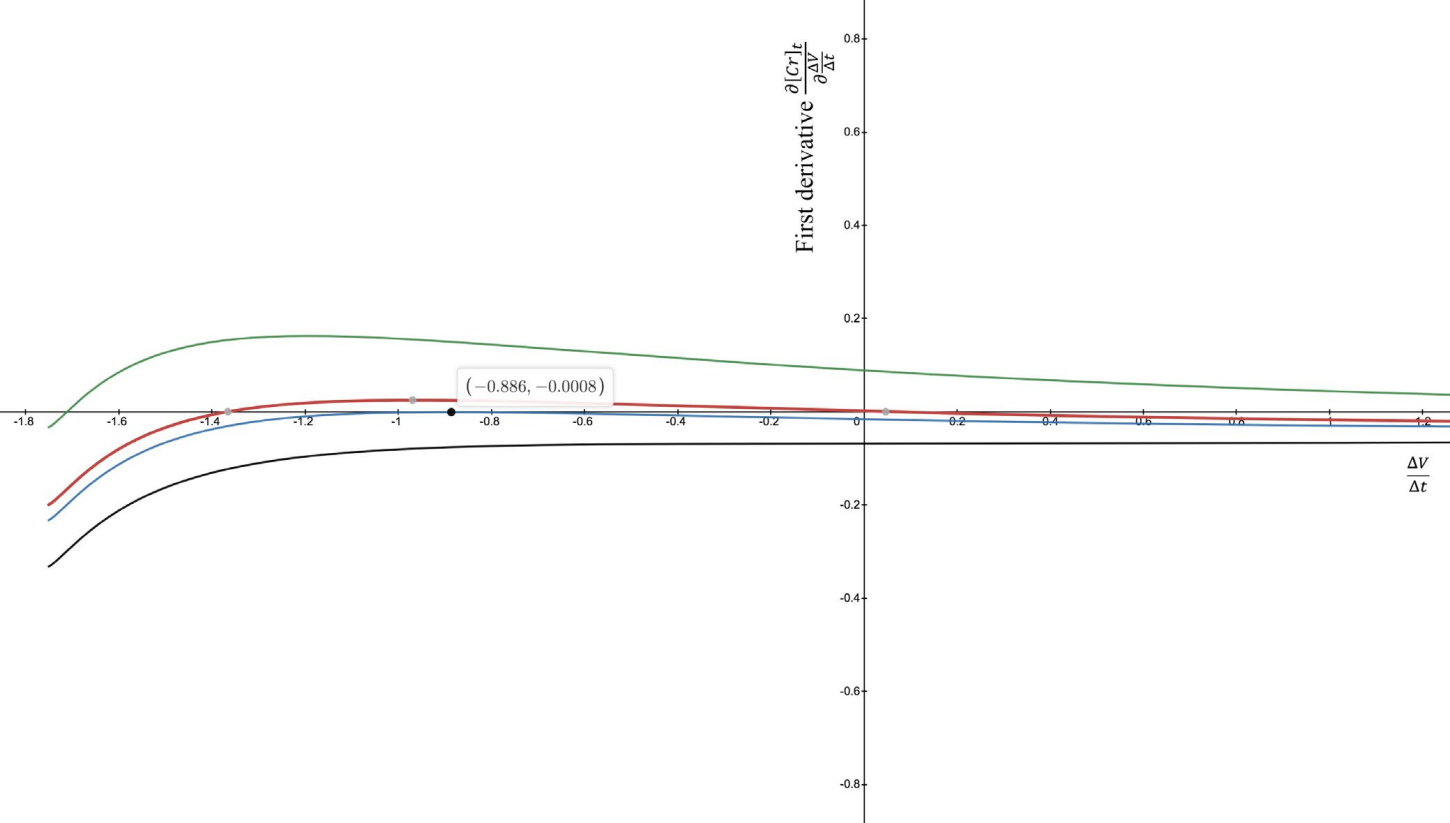
Graphs of first derivative curves when varying only the Gen. Equation (5) is graphed like before. Other variables are $V_{0}=42$ L, t=24 h, ${\mathrm{GFR}}_{K}=100$ mL/min, and ${\left[Cr\right]}_{0}=8$ mg/dL. The Gen is varied between 10 and 100 mg/dL·mL/min. The smallest Gen=10 yields the highest $\frac{\partial {\left[Cr\right]}_{t}}{\partial \frac{\Delta V}{\Delta t}}$ curve (green). As the Gen increases, the curves move downward, until Gen=100 yields the lowest $\frac{\partial {\left[Cr\right]}_{t}}{\partial \frac{\Delta V}{\Delta t}}$ curve (black). Above Gen≈70, the curves are wholly below the x‐axis, meaning that all of their $\frac{\partial {\left[Cr\right]}_{t}}{\partial \frac{\Delta V}{\Delta t}}$ values are negative. But, one other curve (red) is partially above the x‐axis, meaning that some of its $\frac{\partial {\left[Cr\right]}_{t}}{\partial \frac{\Delta V}{\Delta t}}$ values are positive. A positive $\frac{\partial {\left[Cr\right]}_{t}}{\partial \frac{\Delta V}{\Delta t}}$ is promoted by a Gen that is on the smaller side

**FIGURE 3:**
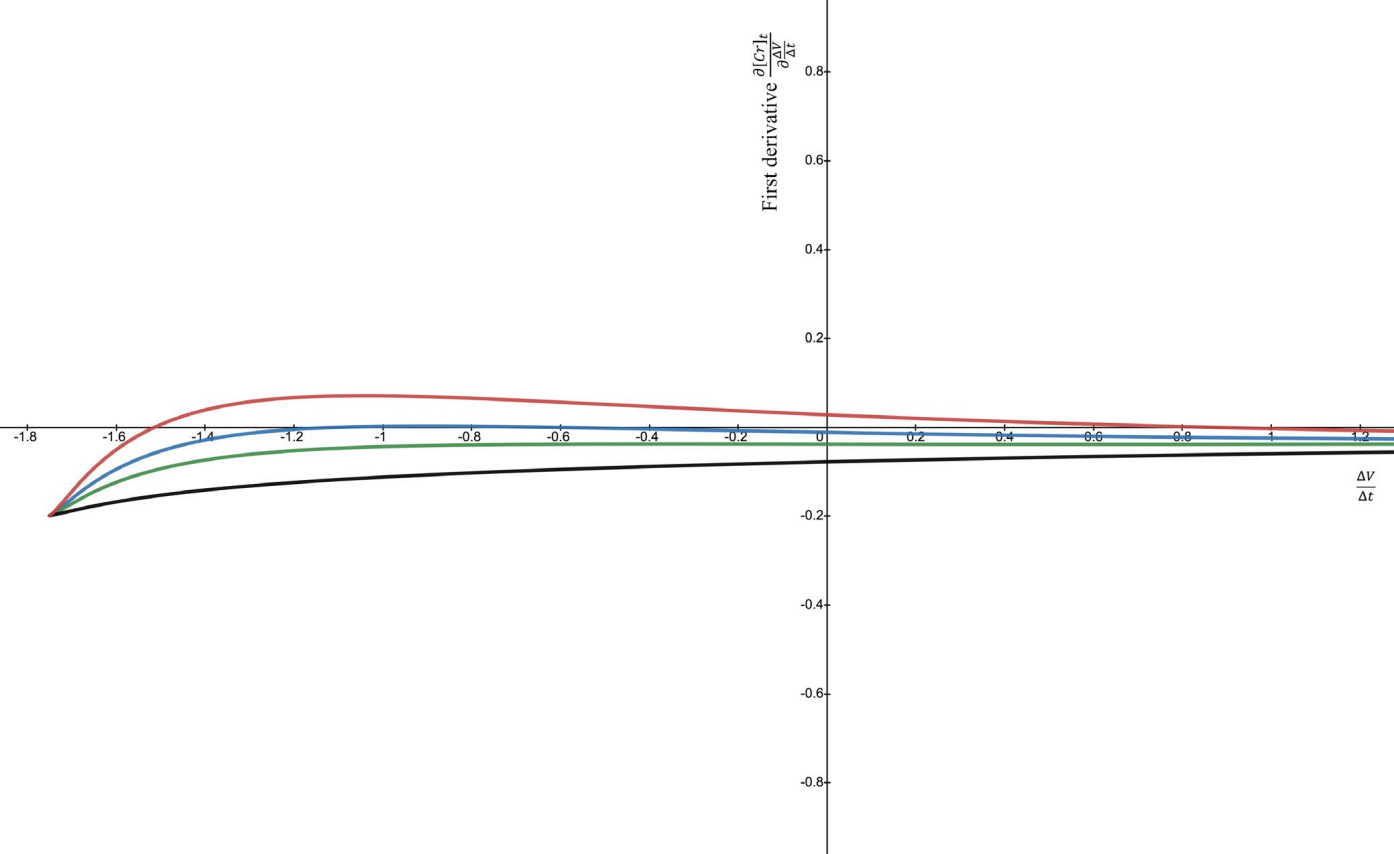
Graphs of first derivative curves when varying only the ${\left[Cr\right]}_{0}$. Like Figure 2, the graphs of the first derivative (y‐axis) vs. $\frac{\Delta V}{\Delta t}$ (x‐axis) shift up or down depending on the initial [creatinine]. The fixed variables are $V_{0}=42$ L, t=24 h, ${\mathrm{GFR}}_{K}=100$ mL/min, Gen=60 mg/dL·mL/min, while the ${\left[Cr\right]}_{0}$ increases from 2 to 5 to 7 to –10 mg/dL. All of the curves are anchored to the same leftmost $\frac{\Delta V}{\Delta t}\left(‐\frac{V_{0}}{t}=‐1.75\;\text{L/h}\right)$ and $\frac{\partial {\left[Cr\right]}_{t}}{\partial \frac{\Delta V}{\Delta t}}$ point. From there, they take different paths with the bottommost curve arising from ${\left[Cr\right]}_{0}=2$ (black) and the uppermost one arising from ${\left[Cr\right]}_{0}=10$ (red). Some curves stay completely below the x‐axis, so their first derivatives are always negative. Some curves rise above the x‐axis for short stretches, after the ${\left[Cr\right]}_{0}$ gets to about 7 (blue), so their first derivatives are positive at times. A positive $\frac{\partial {\left[Cr\right]}_{t}}{\partial \frac{\Delta V}{\Delta t}}$ is fostered by a ${\left[Cr\right]}_{0}$ that is on the larger side

Despite the thought experiment, can $\frac{\partial {\left[Cr\right]}_{t}}{\partial \frac{\Delta V}{\Delta t}}$ become positive under certain conditions? To find out, we varied the parameters and found conditions that work: creatinine generation rate $\left(Gen\right)$ of 60 mg/dl × ml/min and ${\left[Cr\right]}_{0}$ of 8.0 mg/dl that decreases over the next 24 h when the ${\mathrm{GFR}}_{K}$ suddenly increases to 100 ml/min (e.g., by renal replacement therapy) in a patient with a TBW of 42 L. The $\frac{\partial {\left[Cr\right]}_{t}}{\partial \frac{\Delta V}{\Delta t}}$ stays *positive* for most of the negative $\frac{\Delta V}{\Delta t}$ values and for even a few positive $\frac{\Delta V}{\Delta t}$ values (Figure 2, red line, 3 gray dots). Further, decreasing Gen keeps $\frac{\partial {\left[Cr\right]}_{t}}{\partial \frac{\Delta V}{\Delta t}}$ positive for a wider range of $\frac{\Delta V}{\Delta t}$s and makes the $\frac{\partial {\left[Cr\right]}_{t}}{\partial \frac{\Delta V}{\Delta t}}$ peak at a higher positive level (Figure 2, green curve). On the other hand, increasing the Gen lowers the $\frac{\partial {\left[Cr\right]}_{t}}{\partial \frac{\Delta V}{\Delta t}}$ values, until a large enough Gen make the $\frac{\partial {\left[Cr\right]}_{t}}{\partial \frac{\Delta V}{\Delta t}}$ persistently negative for all $\frac{\Delta V}{\Delta t}$ values (Figure 2, blue & black curves). Alternatively, the curves can be moved up or down by varying the ${\left[Cr\right]}_{0}$ (Figure 3). In general, $\frac{\partial {\left[Cr\right]}_{t}}{\partial \frac{\Delta V}{\Delta t}}$ is more likely to be positive if Gen is small and ${\left[Cr\right]}_{0}$ is high; it helps if ${\mathrm{GFR}}_{K}$ is larger and $\frac{\Delta V}{\Delta t}$ is negative. This family of curves has an absolute maximum. If we can find the curve whose maximum lies tangent to y=0, that represents the border between a first derivative being perpetually negative versus potentially positive. In Figure 2, the Gen=70 (blue) curve comes closest to touching y=0. Its maximum $\frac{\partial {\left[Cr\right]}_{t}}{\partial \frac{\Delta V}{\Delta t}}$ is ‐0.0008, but we can place the peak right at 0.

### 3.2 First derivative's peak

To calculate the peak of the $\frac{\partial {\left[Cr\right]}_{t}}{\partial \frac{\Delta V}{\Delta t}}$ vs. $\frac{\Delta V}{\Delta t}$ curve, we differentiated $\frac{\partial {\left[Cr\right]}_{t}}{\partial \frac{\Delta V}{\Delta t}}$ with respect to $\frac{\Delta V}{\Delta t}$ and then set this second derivative equal to zero. Without showing the differentiation steps, we calculated the second derivative to be:

(6) $$\begin{array}{c} \frac{\partial }{\partial \frac{\Delta V}{\Delta t}}\left(\frac{\partial {\left[Cr\right]}_{t}}{\partial \frac{\Delta V}{\Delta t}}\right)=‐{\left(\frac{V_{0}}{V_{0}+\frac{\Delta V}{\Delta t}t}\right)}^{\left(1+\frac{{\mathrm{GFR}}_{K}}{\frac{\Delta V}{\Delta t}}\right)}\cdot \\ \left\{\begin{array}{c} \left[\frac{{\mathrm{GFR}}_{K}}{{\frac{\Delta V}{\Delta t}}^{2}}\cdot \ln\left(\frac{V_{0}}{V_{0}+\frac{\Delta V}{\Delta t}t}\right)+\left(1+\frac{{\mathrm{GFR}}_{K}}{\frac{\Delta V}{\Delta t}}\right)\cdot \frac{t}{V_{0}+\frac{\Delta V}{\Delta t}t}\right]\cdot \left\{\frac{\mathrm{Gen}}{{\left({\mathrm{GFR}}_{K}+\frac{\Delta V}{\Delta t}\right)}^{2}}+\left[\frac{{\mathrm{GFR}}_{K}}{{\frac{\Delta V}{\Delta t}}^{2}}\cdot \ln\left(\frac{V_{0}}{V_{0}+\frac{\Delta V}{\Delta t}t}\right)+\left(1+\frac{{\mathrm{GFR}}_{K}}{\frac{\Delta V}{\Delta t}}\right)\cdot \frac{t}{V_{0}+\frac{\Delta V}{\Delta t}t}\right]\cdot \left(\frac{\mathrm{Gen}}{{\mathrm{GFR}}_{K}+\frac{\Delta V}{\Delta t}}‐{\left[Cr\right]}_{0}\right)\right\}\\ +\left\{\frac{{\mathrm{GFR}}_{K}}{{\frac{\Delta V}{\Delta t}}^{2}}\cdot \left[\frac{2}{\frac{\Delta V}{\Delta t}}\cdot \ln\left(\frac{V_{0}}{V_{0}+\frac{\Delta V}{\Delta t}t}\right)+\frac{t}{V_{0}+\frac{\Delta V}{\Delta t}t}\right]+\left[\frac{{\mathrm{GFR}}_{K}}{{\frac{\Delta V}{\Delta t}}^{2}}+\left(1+\frac{{\mathrm{GFR}}_{K}}{\frac{\Delta V}{\Delta t}}\right)\cdot \frac{t}{V_{0}+\frac{\Delta V}{\Delta t}t}\right]\cdot \frac{t}{V_{0}+\frac{\Delta V}{\Delta t}t}\right\}\cdot \left(\frac{\mathrm{Gen}}{{\mathrm{GFR}}_{K}+\frac{\Delta V}{\Delta t}}‐{\left[Cr\right]}_{0}\right)\\ +\left\{\frac{{\mathrm{GFR}}_{K}}{{\frac{\Delta V}{\Delta t}}^{2}}\cdot \ln\left(\frac{V_{0}}{V_{0}+\frac{\Delta V}{\Delta t}t}\right)+\left(1+\frac{{\mathrm{GFR}}_{K}}{\frac{\Delta V}{\Delta t}}\right)\cdot \frac{t}{V_{0}+\frac{\Delta V}{\Delta t}t}+\left[1‐{\left(\frac{V_{0}}{V_{0}+\frac{\Delta V}{\Delta t}t}\right)}^{‐\left(1+\frac{{\mathrm{GFR}}_{K}}{\frac{\Delta V}{\Delta t}}\right)}\right]\cdot \frac{2}{{\mathrm{GFR}}_{K}+\frac{\Delta V}{\Delta t}}\right\}\cdot \frac{\mathrm{Gen}}{{\left({\mathrm{GFR}}_{K}+\frac{\Delta V}{\Delta t}\right)}^{2}} \end{array}\right\} \end{array}$$

We set $\frac{\partial }{\partial \frac{\Delta V}{\Delta t}}\left(\frac{\partial {\left[Cr\right]}_{t}}{\partial \frac{\Delta V}{\Delta t}}\right)=0$ and solved for $\frac{\Delta V}{\Delta t}$ by Newton's method or the secant method. At one $\frac{\Delta V}{\Delta t}$ root, our example first derivative (Section 3.1, second paragraph) attains its maximum and is positive. On either side of the $\frac{\Delta V}{\Delta t}$ root, the $\frac{\partial {\left[Cr\right]}_{t}}{\partial \frac{\Delta V}{\Delta t}}$ values are decreasing. At the other $\frac{\Delta V}{\Delta t}$ root, our first derivative has a relative minimum. At the left extreme, $\frac{\Delta V}{\Delta t}\leq ‐\frac{V_{0}}{t}$ truncates the plot (Figure 2), because $\frac{\partial {\left[Cr\right]}_{t}}{\partial \frac{\Delta V}{\Delta t}}$ becomes a complex number: in Equation (5), once $V_{0}+\frac{\Delta V}{\Delta t}t$ turns negative, then ${\left(\frac{V_{0}}{V_{0}+\frac{\Delta V}{\Delta t}t}\right)}^{\left(1+\frac{{\mathrm{GFR}}_{K}}{\frac{\Delta V}{\Delta t}}\right)}$ is a negative base raised to a non‐integer power. At the right extreme, $\frac{\Delta V}{\Delta t}\to +\infty$ makes $\frac{\partial {\left[Cr\right]}_{t}}{\partial \frac{\Delta V}{\Delta t}}$ approach zero in the limit. Overall, solving $\frac{\partial }{\partial \frac{\Delta V}{\Delta t}}\left(\frac{\partial {\left[Cr\right]}_{t}}{\partial \frac{\Delta V}{\Delta t}}\right)=0$ yields a single maximum for $\frac{\partial {\left[Cr\right]}_{t}}{\partial \frac{\Delta V}{\Delta t}}$, one that happens to be absolute, and a single minimum for $\frac{\partial {\left[Cr\right]}_{t}}{\partial \frac{\Delta V}{\Delta t}}$, one that is relative.

### 3.3 Making the first derivative's peak tangent to the x‐axis

Setting the second derivative equal to zero optimizes the first derivative, but the first derivative's absolute maximum is not necessarily zero. To find a curve whose maximum is tangent to y=0, we devised a way to make both the first derivative and the second derivative equal to zero at the same time. In doing so, we find the transition to $\frac{\partial {\left[Cr\right]}_{t}}{\partial \frac{\Delta V}{\Delta t}}$ being potentially positive (the Gen=70 curve came close in Figure 2). To solve the simultaneous equations, $\frac{\partial }{\partial \frac{\Delta V}{\Delta t}}\left(\frac{\partial {\left[Cr\right]}_{t}}{\partial \frac{\Delta V}{\Delta t}}\right)=0$ and $\frac{\partial {\left[Cr\right]}_{t}}{\partial \frac{\Delta V}{\Delta t}}=0$, we used algebraic substitution.

In the first and second derivatives [Equations (5) and (6)], only two variables can be explicitly solved for, namely Gen and ${\left[Cr\right]}_{0}$. Set the first derivative equal to zero and solve for ${\left[Cr\right]}_{0}$:

(7) $$\begin{array}{c} {\left[\mathrm{Cr}\right]}_{0}=\frac{\mathrm{Gen}}{GFR_{K}+\frac{\Delta V}{\Delta t}}\\ ‐\frac{\left[1‐{\left(\frac{V_{0}}{V_{0}+\frac{\Delta V}{\Delta t}t}\right)}^{\left(1+\frac{GFR_{K}}{\frac{\Delta V}{\Delta t}}\right)}\right]\cdot \frac{\mathrm{Gen}}{{\left(GFR_{K}+\frac{\Delta V}{\Delta t}\right)}^{2}}}{{\left(\frac{V_{0}}{V_{0}+\frac{\Delta V}{\Delta t}t}\right)}^{\left(1+\frac{GFR_{K}}{\frac{\Delta V}{\Delta t}}\right)}\cdot \left[\frac{GFR_{K}}{{\frac{\Delta V}{\Delta t}}^{2}}\cdot \ln\left(\frac{V_{0}}{V_{0}+\frac{\Delta V}{\Delta t}t}\right)+\left(1+\frac{GFR_{K}}{\frac{\Delta V}{\Delta t}}\right)\cdot \frac{t}{V_{0}+\frac{\Delta V}{\Delta t}t}\right]} \end{array}$$

Substitute this ${\left[Cr\right]}_{0}$, arising from $\frac{\partial {\left[Cr\right]}_{t}}{\partial \frac{\Delta V}{\Delta t}}=0$, in place of the ${\left[Cr\right]}_{0}$ from $\frac{\partial }{\partial \frac{\Delta V}{\Delta t}}\left(\frac{\partial {\left[Cr\right]}_{t}}{\partial \frac{\Delta V}{\Delta t}}\right)=0$. After a lot of algebra, the key to the simultaneous equations $\frac{\partial {\left[Cr\right]}_{t}}{\partial \frac{\Delta V}{\Delta t}}=\frac{\partial }{\partial \frac{\Delta V}{\Delta t}}\left(\frac{\partial {\left[Cr\right]}_{t}}{\partial \frac{\Delta V}{\Delta t}}\right)=0$ is to solve:

(8) $$\langle \begin{array}{c} \left\{\begin{array}{c} 2\left[\frac{{\mathrm{GFR}}_{K}}{{\frac{\Delta V}{\Delta t}}^{2}}\cdot \ln\left(\frac{V_{0}}{V_{0}+\frac{\Delta V}{\Delta t}t}\right)+\left(1+\frac{{\mathrm{GFR}}_{K}}{\frac{\Delta V}{\Delta t}}\right)\cdot \frac{t}{V_{0}+\frac{\Delta V}{\Delta t}t}\right]\cdot \frac{1}{{\mathrm{GFR}}_{K}+\frac{\Delta V}{\Delta t}}\\ ‐\left[2\frac{{\mathrm{GFR}}_{K}}{{\frac{\Delta V}{\Delta t}}^{2}}+\left(1+\frac{{\mathrm{GFR}}_{K}}{\frac{\Delta V}{\Delta t}}\right)\cdot \frac{t}{V_{0}+\frac{\Delta V}{\Delta t}t}\right]\cdot \frac{t}{V_{0}+\frac{\Delta V}{\Delta t}t}\\ ‐2\frac{{\mathrm{GFR}}_{K}}{{\frac{\Delta V}{\Delta t}}^{3}}\cdot \ln\left(\frac{V_{0}}{V_{0}+\frac{\Delta V}{\Delta t}t}\right) \end{array}\right\}\cdot \left[1‐{\left(\frac{V_{0}}{V_{0}+\frac{\Delta V}{\Delta t}t}\right)}^{\left(1+\frac{{\mathrm{GFR}}_{K}}{\frac{\Delta V}{\Delta t}}\right)}\right]\\ ‐{\left[\frac{{\mathrm{GFR}}_{K}}{{\frac{\Delta V}{\Delta t}}^{2}}\cdot \ln\left(\frac{V_{0}}{V_{0}+\frac{\Delta V}{\Delta t}t}\right)+\left(1+\frac{{\mathrm{GFR}}_{K}}{\frac{\Delta V}{\Delta t}}\right)\cdot \frac{t}{V_{0}+\frac{\Delta V}{\Delta t}t}\right]}^{2}\cdot \left[1+{\left(\frac{V_{0}}{V_{0}+\frac{\Delta V}{\Delta t}t}\right)}^{\left(1+\frac{{\mathrm{GFR}}_{K}}{\frac{\Delta V}{\Delta t}}\right)}\right] \end{array}\rangle =0$$

In Equation (8), we supply values for ${\mathrm{GFR}}_{K}$, $V_{0}$, and t and then calculate $\frac{\Delta V}{\Delta t}$ using a root‐finding method. At that $\frac{\Delta V}{\Delta t}$, the peak of the $\frac{\partial {\left[Cr\right]}_{t}}{\partial \frac{\Delta V}{\Delta t}}$ vs. $\frac{\Delta V}{\Delta t}$ curve will touch the x‐axis from below. However, Equation (8) does not contain either Gen or ${\left[Cr\right]}_{0}$. To find those values, we refer back to the first derivative equaling zero. When $\frac{\partial {\left[Cr\right]}_{t}}{\partial \frac{\Delta V}{\Delta t}}=0$, Equation (7) yields ${\left[Cr\right]}_{0}$. We just have to supply a value for Gen and be sure to use the newly calculated $\frac{\Delta V}{\Delta t}$, not the patient's actual $\frac{\Delta V}{\Delta t}$. Or, if ${\left[Cr\right]}_{0}$ is known, as measured by the laboratory, then a rearrangement of $\frac{\partial {\left[Cr\right]}_{t}}{\partial \frac{\Delta V}{\Delta t}}=0$ yields Gen:

(9) $$Gen=\frac{{\left(\frac{V_{0}}{V_{0}+\frac{\Delta V}{\Delta t}t}\right)}^{\left(1+\frac{{\mathrm{GFR}}_{K}}{\frac{\Delta V}{\Delta t}}\right)}\cdot \left[\frac{{\mathrm{GFR}}_{K}}{{\frac{\Delta V}{\Delta t}}^{2}}\cdot \ln\left(\frac{V_{0}}{V_{0}+\frac{\Delta V}{\Delta t}t}\right)+\left(1+\frac{{\mathrm{GFR}}_{K}}{\frac{\Delta V}{\Delta t}}\right)\cdot \frac{t}{V_{0}+\frac{\Delta V}{\Delta t}t}\right]\cdot {\left[Cr\right]}_{0}}{{\left(\frac{V_{0}}{V_{0}+\frac{\Delta V}{\Delta t}t}\right)}^{\left(1+\frac{{\mathrm{GFR}}_{K}}{\frac{\Delta V}{\Delta t}}\right)}\cdot \left[\frac{{\mathrm{GFR}}_{K}}{{\frac{\Delta V}{\Delta t}}^{2}}\cdot \ln\left(\frac{V_{0}}{V_{0}+\frac{\Delta V}{\Delta t}t}\right)+\left(1+\frac{{\mathrm{GFR}}_{K}}{\frac{\Delta V}{\Delta t}}\right)\cdot \frac{t}{V_{0}+\frac{\Delta V}{\Delta t}t}\right]\cdot \frac{1}{{\mathrm{GFR}}_{K}+\frac{\Delta V}{\Delta t}}‐\left[1‐{\left(\frac{V_{0}}{V_{0}+\frac{\Delta V}{\Delta t}t}\right)}^{\left(1+\frac{{\mathrm{GFR}}_{K}}{\frac{\Delta V}{\Delta t}}\right)}\right]\cdot \frac{1}{{\left({\mathrm{GFR}}_{K}+\frac{\Delta V}{\Delta t}\right)}^{2}}}$$

### 3.4 Testing if the peak is tangent to the x‐axis

Equation (8) reveals how $\frac{\partial {\left[Cr\right]}_{t}}{\partial \frac{\Delta V}{\Delta t}}$ at its maximum can equal zero. From Figure 2, plug ${\mathrm{GFR}}_{K}=100$ ml/min, $V_{0}=42$ L, and t=24 h into Equation (8). Use a root‐finding method to determine that $\frac{\Delta V}{\Delta t}=‐0.88928$… L/h. Figure 2 had a uniform ${\left[Cr\right]}_{0}$ of 8 mg/dL. Plug that into Equation (9) to find that Gen=69.67084… This is the value, not Gen=70, that places the $\frac{\partial {\left[Cr\right]}_{t}}{\partial \frac{\Delta V}{\Delta t}}$’s absolute maximum on the x‐axis, exactly. Alternatively, plug Gen=70 into Equation (7) to find that a ${\left[Cr\right]}_{0}=8.03779$… would have also placed the curve's peak on the x‐axis. Any combination, really, of Gen and ${\left[Cr\right]}_{0}$ would work as long as the $\frac{\mathrm{Gen}}{{\left[Cr\right]}_{0}}$ ratio is $\frac{69.67084\cdots }{8}=\frac{70}{8.03779\cdots }=8.708$… ml/min (in this case). Broadly, the $\frac{\mathrm{Gen}}{{\left[Cr\right]}_{0}}$ ratio is a fixed attribute for a set of ${\mathrm{GFR}}_{K}$, $V_{0}$, and t inputs that allows the first and second derivatives to equal zero simultaneously.

### 3.5 Gen and ${\left[Cr\right]}_{0}$ effects: lifting the peak into positive territory

Now that the peak can be positioned at the x‐axis, how can the $\frac{\partial {\left[Cr\right]}_{t}}{\partial \frac{\Delta V}{\Delta t}}$ be lifted above the x‐axis? The ${\mathrm{GFR}}_{K}$, $V_{0}$, and t are initial data, and $\frac{\Delta V}{\Delta t}$ is the independent variable on the graph. That leaves only Gen and ${\left[Cr\right]}_{0}$ to be manipulated. Using the fixed $\frac{\mathrm{Gen}}{{\left[Cr\right]}_{0}}$ ratio as a benchmark, we find that lower ratios shift the curve partially into positive territory, in keeping with the observation that smaller Gens and/or bigger ${\left[Cr\right]}_{0}$s promote $\frac{\partial {\left[Cr\right]}_{t}}{\partial \frac{\Delta V}{\Delta t}}$ being positive. In practice, one can calculate ${\left[Cr\right]}_{0}$ by Equation (7), for example, and then ask if the patient's actual ${\left[Cr\right]}_{0}$ is larger, which lets $\frac{\partial {\left[Cr\right]}_{t}}{\partial \frac{\Delta V}{\Delta t}}$ be positive at times. Or, one can calculate the benchmark Gen by Equation (9) and then ask if the patient's actual Gen is smaller, which also permits $\frac{\partial {\left[Cr\right]}_{t}}{\partial \frac{\Delta V}{\Delta t}}$ to be positive.

### 3.6 ${\mathrm{GFR}}_{K}$ effect: variant way for $\frac{\partial {\left[Cr\right]}_{t}}{\partial \frac{\Delta V}{\Delta t}}$ to be positive

In Figures 2 and 3, the stereotypical shape of the $\frac{\partial {\left[Cr\right]}_{t}}{\partial \frac{\Delta V}{\Delta t}}$ vs. $\frac{\Delta V}{\Delta t}$ curve, from left to right, is that $\frac{\partial {\left[Cr\right]}_{t}}{\partial \frac{\Delta V}{\Delta t}}$ rises from a negative value to peak at an absolute maximum which can be positive, then falls to a relative minimum (mentioned in Section 3.2) that is negative, and then asymptotically increases toward y=0. The curve is shifted vertically, more or less, by varying the Gen or ${\left[Cr\right]}_{0}$. Well, the curve is shifted horizontally, mostly, by varying the ${\mathrm{GFR}}_{K}$. A higher ${\mathrm{GFR}}_{K}$ pulls the curve rightward, and a lower ${\mathrm{GFR}}_{K}$ pushes it leftward. Also, imagine that the left end of the curve is tethered to an invisible wall at $\frac{\Delta V}{\Delta t}=‐\frac{V_{0}}{t}$ but has the ability to slide up or down that wall. Then, a right shift would stretch the curve, flattening it out, and a left shift would compress the curve, bunching it up against the wall in an orderly way by making it bend and stack in layers (with no thickness). Can the ${\mathrm{GFR}}_{K}$ be lowered sufficiently to left‐shift the absolute maximum until it is located at the leftmost $\frac{\Delta V}{\Delta t}$, that is, $‐\frac{V_{0}}{t}$? Going further, can the left shift continue until the relative minimum is then pressed up against the leftmost $\frac{\Delta V}{\Delta t}$ wall? If so, these max/min at the leftmost $\frac{\Delta V}{\Delta t}$ would correspond to a second derivative equaling zero at two ${\mathrm{GFR}}_{K}$ roots, one for the max and one for the min.

**FIGURE 4:**
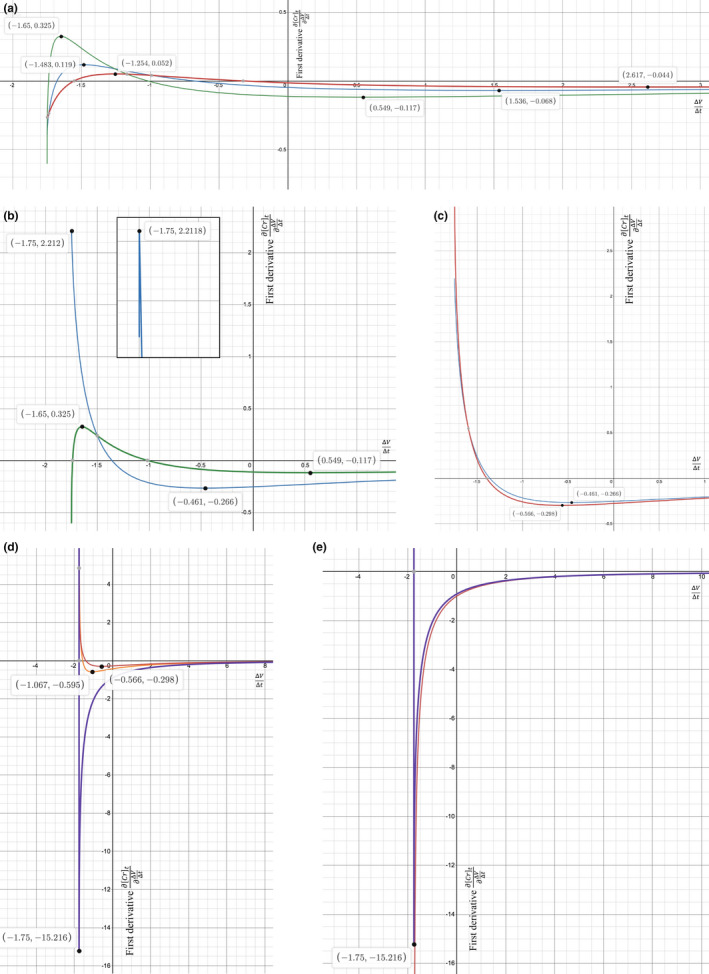
Decreasing ${\mathrm{GFR}}_{K}$ pushes the first derivative curve leftward along the $\frac{\Delta V}{\Delta t}$ x‐axis. Variables in common are Gen=60 mg/dL·mL/min, ${\left[Cr\right]}_{0}=8$ mg/dL, $V_{0}=42$ L, and t=24 h. (a) As the ${\mathrm{GFR}}_{K}$ decreases from 90 (red) to 80 (blue) to 70 (green) mL/min, the curve looks like it is being pushed to the left and is bending in the process. The maximum moves steadily up, the minimum moves down, and both of them move to the left. Also, the left endpoint slides down a virtual wall at the leftmost $\frac{\Delta V}{\Delta t}$. (b) When ${\mathrm{GFR}}_{K}$ decreases to 58.34 $\left(≅2\frac{V_{0}}{t}\right)$, the maximum has been pushed to the leftmost $\frac{\Delta V}{\Delta t}$, and only a short tail to the left of the maximum is decreasing before it gets truncated at the wall (inset). (c) As ${\mathrm{GFR}}_{K}$ decreases below 58.34, the maximum vanishes (blue) and transitions into a left tail that blows up to +∞ (red). (d) With no more maximum, the minimum is the sole critical point, and it continues to move down and left as the ${\mathrm{GFR}}_{K}$ decreases further from 57 (red) to 50 (orange) mL/min. When the ${\mathrm{GFR}}_{K}$ drops to 36.67 $\left(≅\frac{\mathrm{Gen}}{{\left[Cr\right]}_{0}}+\frac{V_{0}}{t}\right)$, the minimum has been pushed to the leftmost $\frac{\Delta V}{\Delta t}$, and the left tail still diverges to +∞ (purple). (e) When ${\mathrm{GFR}}_{K}$ decreases below 36.67, the minimum vanishes (purple) and transitions into a left tail that plunges to ‐∞ (red). From here, the first derivative is always negative

As ${\mathrm{GFR}}_{K}$ is reduced, the curve acts like a rope being pushed leftward against a wall, based on tracking the maximum and minimum $\frac{\partial {\left[Cr\right]}_{t}}{\partial \frac{\Delta V}{\Delta t}}$ points and the sliding along the wall. In response to the push, the endpoint at the leftmost $\frac{\Delta V}{\Delta t}$ moves down, the maximum moves up, and the minimum moves down, like how a rope could fold to be more compact (Figure 4a). In addition to the vertical motions, the max/min points move horizontally to the left. Once the ${\mathrm{GFR}}_{K}$ is lowered to ~58.34 (in this example), the bend at the maximum is very sharp and the maximum is left‐shifted all the way to $\frac{\Delta V}{\Delta t}≅‐\frac{V_{0}}{t}$ (Figure 4b). As the ${\mathrm{GFR}}_{K}$ is lowered some more, the minimum continues to move down and left but the absolute maximum is transitioned into the left endpoint of the curve sliding *up* the wall, on its way to +∞ (Figure 4c). In this way, certain ${\mathrm{GFR}}_{K}$s can enforce a positive $\frac{\partial {\left[Cr\right]}_{t}}{\partial \frac{\Delta V}{\Delta t}}$.

### 3.7 ${\mathrm{GFR}}_{K}$ effect: keeping $\frac{\partial {\left[Cr\right]}_{t}}{\partial \frac{\Delta V}{\Delta t}}$ negative

As ${\mathrm{GFR}}_{K}$ is further reduced, with no more sliding down the wall for now, the relative minimum becomes an absolute minimum (Figure 4c). As the ${\mathrm{GFR}}_{K}$ reduction keeps pushing the curve/rope to the left against a wall, the bend gets sharper and the minimum moves even more to the left and down. When the ${\mathrm{GFR}}_{K}$ gets down to ~36.67 (in this example), the minimum is left‐shifted all the way to the leftmost $\frac{\Delta V}{\Delta t}$ (Figure 4d), like the maximum was earlier. As ${\mathrm{GFR}}_{K}$ is lowered past ~36.67, the absolute minimum is transitioned into the left endpoint of the curve sliding *down* the wall, on its way to ‐∞ (Figure 4e). After this transition, the $\frac{\partial {\left[Cr\right]}_{t}}{\partial \frac{\Delta V}{\Delta t}}$ will always be negative.

For details on how the kinetic GFR can alter the shape of the first derivative curve and help determine whether $\frac{\partial {\left[Cr\right]}_{t}}{\partial \frac{\Delta V}{\Delta t}}$ can be positive, please see the Appendix.

### 3.8 Algorithm to determine if $\frac{\partial {\left[Cr\right]}_{t}}{\partial \frac{\Delta V}{\Delta t}}$ can be positive

If all variables have allowable values $\left(V_{0},V_{0}+\frac{\Delta V}{\Delta t}t,t,{\mathrm{GFR}}_{K},Gen,\;{\left[Cr\right]}_{0}\;\text{all non‐negative}\right)$, one way to detect potential positivity of $\frac{\partial {\left[Cr\right]}_{t}}{\partial \frac{\Delta V}{\Delta t}}$ is to compile the lessons above into an algorithm. If the ${\mathrm{GFR}}_{K}$ roots are in a “permissive” order of $\frac{\mathrm{Gen}}{{\left[Cr\right]}_{0}}+\frac{V_{0}}{t}<2\frac{V_{0}}{t}$, permitting $\frac{\partial {\left[Cr\right]}_{t}}{\partial \frac{\Delta V}{\Delta t}}$ to be positive, then:

- For $0\leq {\mathrm{GFR}}_{K}<\frac{\mathrm{Gen}}{{\left[Cr\right]}_{0}}+\frac{V_{0}}{t}$ (bottom domain), the first derivative will always be negative. See A1.3.
- For $\frac{\mathrm{Gen}}{{\left[Cr\right]}_{0}}+\frac{V_{0}}{t}<{\mathrm{GFR}}_{K}<2\frac{V_{0}}{t}$ (middle domain), the first derivative has an absolute minimum and the left‐sided tail can diverge to +∞ at the leftmost $\frac{\Delta V}{\Delta t}$ $\left(\to ‐\frac{V_{0}}{t}\right)$, with exceptions (see A1.2).
- For $2\frac{V_{0}}{t}<{\mathrm{GFR}}_{K}$ (top domain), the first derivative has an absolute maximum, which *can* be positive (see Section 3.2).
  - Calculate by a root‐finding method the $\frac{\Delta V}{\Delta t}$ at which the $\frac{\partial {\left[Cr\right]}_{t}}{\partial \frac{\Delta V}{\Delta t}}$ vs. $\frac{\Delta V}{\Delta t}$ curve is tangent to the x‐axis at its absolute maximum, that is, solve Equation (8) for $\frac{\Delta V}{\Delta t}$ (see Section 3.3).
  - Plug that $\frac{\Delta V}{\Delta t}$ and a known Gen into Equation (7) to calculate a benchmark ${\left[Cr\right]}_{0}$ (Section 3.5).
    1. If the patient's ${\left[Cr\right]}_{0}$ is greater than the benchmark ${\left[Cr\right]}_{0}$, then the absolute maximum lies above the x‐axis and $\frac{\partial {\left[Cr\right]}_{t}}{\partial \frac{\Delta V}{\Delta t}}$ can be positive.
    2. If the patient's ${\left[Cr\right]}_{0}$ is less than the benchmark ${\left[Cr\right]}_{0}$, then the absolute maximum lies below the x‐axis and $\frac{\partial {\left[Cr\right]}_{t}}{\partial \frac{\Delta V}{\Delta t}}$ is always negative.
  - Alternatively, plug the $\frac{\Delta V}{\Delta t}$ from step c., i. and a known ${\left[Cr\right]}_{0}$ into Equation (9) to calculate a benchmark Gen (see Section 3.5).
    1. If the patient's Gen is less than the benchmark Gen, then the absolute maximum lies above the x‐axis and $\frac{\partial {\left[Cr\right]}_{t}}{\partial \frac{\Delta V}{\Delta t}}$ can be positive.
    2. If the patient's Gen is greater than the benchmark Gen, then the absolute maximum lies below the x‐axis and $\frac{\partial {\left[Cr\right]}_{t}}{\partial \frac{\Delta V}{\Delta t}}$ is always negative.

To know if $\frac{\partial {\left[Cr\right]}_{t}}{\partial \frac{\Delta V}{\Delta t}}$ is positive at the patient's *actual* $\frac{\Delta V}{\Delta t}$, not the calculated $\frac{\Delta V}{\Delta t}$ above, plug all of the patient's variables into Equation (5) and note the sign. One can also find the spread of $\frac{\Delta V}{\Delta t}$ values that yield a positive $\frac{\partial {\left[Cr\right]}_{t}}{\partial \frac{\Delta V}{\Delta t}}$ by a root‐finding method. Vary the initial guess to find both $\frac{\Delta V}{\Delta t}$ roots.

## 4 DISCUSSION

### 4.1 Positive paradox possible?

The positive $\frac{\partial {\left[Cr\right]}_{t}}{\partial \frac{\Delta V}{\Delta t}}$ paradox is fostered by the combination of a low creatinine generation rate and a high initial creatinine. The two conditions are not mutually exclusive but they are at odds with one another, making the combination rare but not impossible. For a low Gen to be paired with a high ${\left[Cr\right]}_{0}$, renal failure probably had to be sustained for a while. To permit $\frac{\partial {\left[Cr\right]}_{t}}{\partial \frac{\Delta V}{\Delta t}}$ to be positive, the ${\mathrm{GFR}}_{K}$ has to be at least $>\frac{\mathrm{Gen}}{{\left[Cr\right]}_{0}}+\frac{V_{0}}{t}$ and preferably $>2\frac{V_{0}}{t}$. The relatively high ${\mathrm{GFR}}_{K}$ is going to decrease the [creatinine] over time $\left({\left[Cr\right]}_{t}\right)$. Though it is decreased overall, can ${\left[Cr\right]}_{t}$ decrease less due to a volume rate increase? Then the ${\left[Cr\right]}_{t}$ would be comparatively increased. Or, can ${\left[Cr\right]}_{t}$ decrease more due to a volume rate decrease? Then the ${\left[Cr\right]}_{t}$ would be comparatively decreased. Either scenario is compatible with a $\frac{\partial {\left[Cr\right]}_{t}}{\partial \frac{\Delta V}{\Delta t}}$ that is positive in sign. But what kind of patient fits the criteria of low Gen, high ${\left[Cr\right]}_{0}$, and a relatively high ${\mathrm{GFR}}_{K}$? One plausible patient may have suffered sepsis that temporarily reduced the Gen (Doi et al., 2009; Prowle et al., 2014). Sepsis may have also caused kidney failure, so the [creatinine] went fairly high. Doctors then initiated continuous renal replacement therapy (CRRT) that provided a ${\mathrm{GFR}}_{K}$ greater than $2\frac{V_{0}}{t}$. (${\mathrm{GFR}}_{K}$ here is not used in the literal sense of clearance done by the glomerulus. Rather, it is used in the broader sense of clearance done by any means, including extracorporeal).

### 4.2 Paradox by the numbers

The abstract math may be easier to grasp if we put some concrete numbers on it. Suppose that a septic patient now has a Gen=40 mg/dl × ml/min. He develops acute tubular necrosis and the creatinine rises to 8 mg/dl. CRRT is started, and the total ${\mathrm{GFR}}_{K}=80$ ml/min. The combination of conditions seems ripe for a positive $\frac{\partial {\left[Cr\right]}_{t}}{\partial \frac{\Delta V}{\Delta t}}$, so the algorithm is consulted. First, the ${\mathrm{GFR}}_{K}$ falls into the top domain, since it is $>2\frac{V_{0}}{t}=\frac{50}{3}\cdot 2\cdot \frac{42}{24}=58.\bar{3}$, assuming his volume (TBW) is 42 L and the time interval is going to be 24 h. The top domain implies that the $\frac{\partial {\left[Cr\right]}_{t}}{\partial \frac{\Delta V}{\Delta t}}$ vs. $\frac{\Delta V}{\Delta t}$ curve will have an absolute maximum. To know where the maximum is tangent to the x‐axis, Equation (8) is solved by a root‐finding method to yield a $\frac{\Delta V}{\Delta t}=‐1.43818$… Plug that $\frac{\Delta V}{\Delta t}$ and the ${\left[Cr\right]}_{0}=8$ into Equation (9) to calculate a benchmark Gen of 78.42… (Alternatively, plug that $\frac{\Delta V}{\Delta t}$ and the Gen=40 into Equation (7) to calculate a benchmark ${\left[Cr\right]}_{0}$ of 4.08…) The patient's Gen of 40 is less than the benchmark Gen, so the absolute maximum lies above the x‐axis. (Alternatively, the patient's ${\left[Cr\right]}_{0}$ of 8 is greater than the benchmark ${\left[Cr\right]}_{0}$, and again the absolute maximum lies above the x‐axis). If the maximum is positive, then $\frac{\partial {\left[Cr\right]}_{t}}{\partial \frac{\Delta V}{\Delta t}}$ stays positive over a spread of $\frac{\Delta V}{\Delta t}$ values. The $\left(+\right)$ sign says that changes in $\frac{\Delta V}{\Delta t}$ move in the same direction as changes in ${\left[Cr\right]}_{t}$.

### 4.3 Effect size

Say that the CRRT ultrafiltration (UF)—volume removal—rate is turned up from 100 to 300 ml/h, that is, the $\frac{\Delta V}{\Delta t}$ goes from ‐0.1 to ‐0.3 L/h, making the $\partial \frac{\Delta V}{\Delta t}$ *negative*. At those two $\frac{\Delta V}{\Delta t}$s, the first derivative is positive (≈0.012 to 0.029 mg/dl per L/h). That forces the $\partial {\left[Cr\right]}_{t}$ to be negative. By Equation (2), the ${\left[Cr\right]}_{t=24}$ goes from 0.982… to 0.978… mg/dl, a decrease that represents a negative $\partial {\left[Cr\right]}_{t}$ as advertised. Certainly, the change in [creatinine] is small, as predicted by the small $\frac{\partial {\left[Cr\right]}_{t}}{\partial \frac{\Delta V}{\Delta t}}$. Importantly, the positive sign assures the nephrologist that turning up the UF rate will actually improve the next day's [creatinine]. One might posit that the ${\left[Cr\right]}_{t}$ improvement is due to the higher UF rate increasing convective clearance (Tandukar & Palevsky, 2019), but the math disproves that by holding the ${\mathrm{GFR}}_{K}$ constant. Besides, turning up the UF rate will *worsen* the next day's [creatinine] if the $\frac{\partial {\left[Cr\right]}_{t}}{\partial \frac{\Delta V}{\Delta t}}$ is negative, so convective clearance does not always match with the [creatinine] trajectory.

### 4.4 Come‐from‐behind win: getting to a lower [creatinine]

**FIGURE 5:**
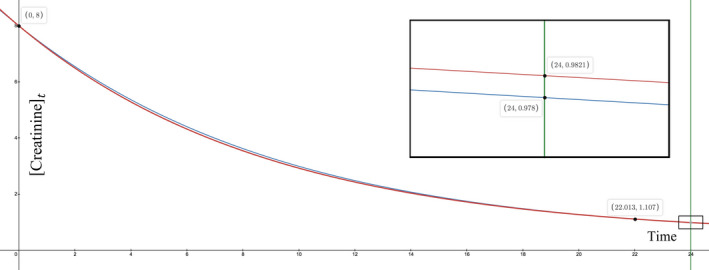
Positive derivative paradox viewed as evolution of [creatinine]. This paradox can happen in a septic patient with kidney failure on continuous dialysis. Raise the ultrafiltration rate from $\frac{\Delta V}{\Delta t}=‐0.1$ to ‐0.3 L/h, which is a negative $\partial \frac{\Delta V}{\Delta t}$, and if the first derivative is positive, then tomorrow's [creatinine] will be further decreased, which is a negative $\partial {\left[Cr\right]}_{24\;h}$. The fixed variables are $V_{0}=42$ L, Gen=40 mg/dL·mL/min, ${\left[Cr\right]}_{0}=8$ mg/dL, and ${\mathrm{GFR}}_{K}=80$ mL/min. Then, Equation (2) is graphed as ${\left[Cr\right]}_{t}$ (y‐axis) versus time (x‐axis). Seen as the evolution of ${\left[Cr\right]}_{t}$, the red curve shows the effect of a baseline $\frac{\Delta V}{\Delta t}=‐0.1$ L/h, while the blue curve shows the effect of a $\frac{\Delta V}{\Delta t}=‐0.3$ L/h. Predictably, both [creatinine] curves decrease over time due to the relatively high ${\mathrm{GFR}}_{K}$ of 80 mL/min. But the blue curve declines more slowly, because its greater volume removal will concentrate the ${\left[Cr\right]}_{t}$ more. As time goes by, the blue curve catches up to the red curve at about 22 h. After that, blue surpasses red and gets to a lower ${\left[Cr\right]}_{t}$ at the 24‐h mark (see inset), consistent with the first derivative being positive

By itself, volume loss should concentrate and thereby increase the [creatinine]. Somehow, this concentration effect is overridden by a creatinine‐lowering effect. In 24 h, $\frac{\Delta V}{\Delta t}=‐0.3$ L/h got to a lower [creatinine] than $\frac{\Delta V}{\Delta t}=‐0.1$ L/h. Having $\frac{\Delta V}{\Delta t}=‐0.3$ L/h would seem like a handicap, because removing more volume concentrates the [creatinine] and resists the ${\mathrm{GFR}}_{K}$ that is trying to lower the [creatinine]. Thus, the $\frac{\Delta V}{\Delta t}=‐0.3$ (Figure 5, blue curve) has a higher [creatinine] than the $\frac{\Delta V}{\Delta t}=‐0.1$ (Figure 5, red curve) at almost all time points. After about 5.7 h, however, the blue curve starts to catch up to the red curve (Figure 5), which is peculiar as the two $\frac{\Delta V}{\Delta t}$s have not changed. Apparently, concentrating the [creatinine] can be advantageous when the higher ${\left[Cr\right]}_{t}$ interacts with the steady ${\mathrm{GFR}}_{K}$ to excrete more creatinine mass. That lowers the total creatinine (numerator) faster than its volume (denominator), such that the creatinine *concentration* starts to decline more quickly. The blue curve catches up to the red curve at ~22 h (Figure 5). Then, the blue curve barely edges out the red curve at the 24‐h mark (Figure 5, see inset), meaning that the higher UF rate $\left(‐0.3\right)$ came from behind to get to a lower [creatinine]. Despite the concentration disadvantage for most of the race, the higher UF rate's latent factor that slowly predominated was a synergy between the ${\left[Cr\right]}_{t}$ and the ${\mathrm{GFR}}_{K}$ to boost creatinine excretion.

### 4.5 Volume gain can increase the [creatinine]

In the same clinical example, the $\frac{\partial {\left[Cr\right]}_{t}}{\partial \frac{\Delta V}{\Delta t}}$ stays positive briefly into the positive $\frac{\Delta V}{\Delta t}$ zone. If volume is given $\left(\frac{\Delta V}{\Delta t}\;\text{positive}\right)$, could that increase the [creatinine]? Yes. If the UF is turned off and CRRT is used to give volume, let us say that $\frac{\Delta V}{\Delta t}$ increases from ‐100 to 80 ml/h. The $\partial \frac{\Delta V}{\Delta t}$ is certainly positive. The $\frac{\partial {\left[Cr\right]}_{t}}{\partial \frac{\Delta V}{\Delta t}}$ remains positive. That forces $\partial {\left[Cr\right]}_{t}$ to be positive too. In a race between $\frac{\Delta V}{\Delta t}=‐0.1$ and +0.08 L/h, the [creatinine] at 24 h is 0.982… vs. 0.983… mg/dl, respectively. Counterintuitively, giving volume resulted in a *higher* [creatinine] than continuing the UF. The explanation is similar to before. The baseline UF rate $\left(\frac{\Delta V}{\Delta t}=‐0.1\right)$, by virtue of the concentration effect, lags behind in lowering the ${\left[Cr\right]}_{t}$. Meanwhile, volume gain $\left(\frac{\Delta V}{\Delta t}=+0.08\right)$ is diluting the ${\left[Cr\right]}_{t}$ and helping the ${\mathrm{GFR}}_{K}$. Because the UF has a higher ${\left[Cr\right]}_{t}$ that is subjected to a relatively high ${\mathrm{GFR}}_{K}$ for most of the race, more creatinine is excreted that eventually lowers the [creatinine] further versus a gain of volume, even with the latter's dilution effect advantage. So, the creatinine‐lowering effect that overcomes the volume effect is facilitated by a higher ${\mathrm{GFR}}_{K}$, which explains why the ${\mathrm{GFR}}_{K}$ should be $>2\frac{V_{0}}{t}$ to get a positive $\frac{\partial {\left[Cr\right]}_{t}}{\partial \frac{\Delta V}{\Delta t}}$.

### 4.6 Reality check

What if the ${\mathrm{GFR}}_{K}$ is in the middle domain (see Section 3.8, b.)? That gives the $\frac{\partial {\left[Cr\right]}_{t}}{\partial \frac{\Delta V}{\Delta t}}$ curve an absolute minimum, and the tail to the left can be positive, maybe even going to +∞. Unfortunately, obtaining a positive first derivative this way is clinically unrealistic. The $\frac{\Delta V}{\Delta t}$ is usually so negative that it would dry up nearly all of the TBW within an allotted time, killing the patient. Realistically, all of the positive first derivatives in medicine come from a ${\mathrm{GFR}}_{K}$ being in the top domain of $>2\frac{V_{0}}{t}$.

### 4.7 Big picture

A positive $\frac{\partial {\left[Cr\right]}_{t}}{\partial \frac{\Delta V}{\Delta t}}$ paradox may not happen all that often, but it is a real mathematical phenomenon that can occur under the right circumstances, especially in septic patients who have become quite azotemic and are being initiated on CRRT, a not uncommon scenario. In those cases, clinicians may want to pay attention to the CRRT volume settings. Turning up the UF rate, that is, decreasing the $\frac{\Delta V}{\Delta t}$, can lower the ${\left[Cr\right]}_{t}$ a little more. On the other hand, turning down the UF rate (or giving volume), that is, increasing the $\frac{\Delta V}{\Delta t}$, can raise the ${\left[Cr\right]}_{t}$ a little. This counterintuitive improvement or worsening of [creatinine] is marginal at best and pales in comparison to the overall effect that CRRT exerts on the [creatinine] trajectory. In addition, the paradox goes unnoticed because one patient cannot experience two separate $\frac{\Delta V}{\Delta t}$ rates to yield two ${\left[Cr\right]}_{t}$s for comparison.

Most patients will not be at risk for a positive paradox. The combination of low Gen, high ${\left[Cr\right]}_{0}$, and high‐ish ${\mathrm{GFR}}_{K}$ is rare. The ${\mathrm{GFR}}_{K}$ is $<\frac{\mathrm{Gen}}{{\left[Cr\right]}_{0}}+\frac{V_{0}}{t}$ in many instances of AKI, so those patients are protected from a paradox and likely will behave as expected in response to fluids or diuresis. Milder cases of AKI can have a ${\mathrm{GFR}}_{K}$ that lies between $\frac{\mathrm{Gen}}{{\left[Cr\right]}_{0}}+\frac{V_{0}}{t}$ and $2\frac{V_{0}}{t}$, which does permit a paradox but only under a ludicrous rate of volume loss $\left(\frac{\Delta V}{\Delta t}\to ‐\frac{V_{0}}{t}\right)$ that is clinically unrealistic. If the ${\mathrm{GFR}}_{K}$ is high‐ish enough to be $>2\frac{V_{0}}{t}$, the paradox, if it occurs, alters ${\left[Cr\right]}_{t}$ in a negligible way. Finally, more than just the $\frac{\Delta V}{\Delta t}$ changes in clinical practice, so if the paradox seems to occur, it may be due to the other variables changing and confounding the picture. With all that said, we think the possibility of a positive $\frac{\partial {\left[Cr\right]}_{t}}{\partial \frac{\Delta V}{\Delta t}}$ is intellectually enlightening, and it differs markedly from the $\frac{\partial {\left[Cr\right]}_{t}}{\partial {\mathrm{GFR}}_{K}}$ that was proved to always be negative (Chen & Chiaramonte, 2021).

## CONFLICT OF INTEREST

The authors have no conflicts of interest.

## AUTHOR CONTRIBUTION

Sheldon Chen: conception and design of study, mathematical derivations and equation graphs, interpretation of data, writing and revision of manuscript, and final approval of the manuscript. Robert Chiaramonte: confirmation of mathematical derivations and equation graphs, interpretation of data, revision, and final approval of the manuscript.

## Figures and Tables

**FIGURE A1:**
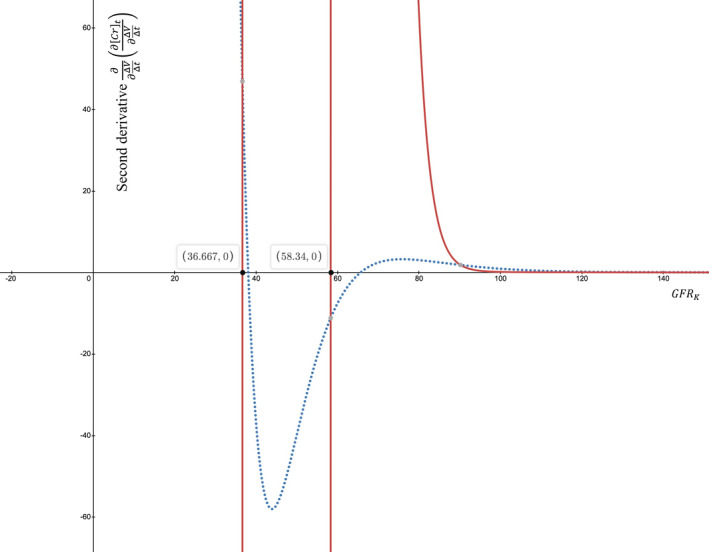
Second derivative goes to infinity near the leftmost $\frac{\Delta V}{\Delta t}$ but still has two ${\mathrm{GFR}}_{K}$ roots. Equation (6) is graphed with ${\mathrm{GFR}}_{K}$ as an independent variable (x‐axis) and $\frac{\partial }{\partial \frac{\Delta V}{\Delta t}}\left(\frac{\partial {\left[Cr\right]}_{t}}{\partial \frac{\Delta V}{\Delta t}}\right)$ as the dependent variable (y‐axis). The fixed variables in this figure are $V_{0}=42$ L, t=24 h, Gen=60 mg/dL·mL/min, and ${\left[Cr\right]}_{0}=8$ mg/dL, while the two $\frac{\Delta V}{\Delta t}$ values are ‐1.74999 $\left(\text{approaching}\;‐\frac{V_{0}}{t}\right)$ and ‐1.7 L/h. For $\frac{\Delta V}{\Delta t}$ at its leftmost, the second derivative is volatile. It swings to and from ±∞ (red curve). Yet, it crosses the x‐axis twice and therefore has two roots that can be estimated as ${\mathrm{GFR}}_{K}\approx \frac{\mathrm{Gen}}{{\left[Cr\right]}_{0}}+\frac{V_{0}}{t}=36.\bar{6}$ and ${\mathrm{GFR}}_{K}\approx 2\frac{V_{0}}{t}=58.\bar{3}$. As $\frac{\Delta V}{\Delta t}$ is increased, the second derivative curve shifts to the right (blue dot curve). It is not as volatile, and its roots are greater than when $\frac{\Delta V}{\Delta t}$ is leftmost. In a way, the ${\mathrm{GFR}}_{K}$ roots are a continuum, beginning at $\frac{\mathrm{Gen}}{{\left[Cr\right]}_{0}}+\frac{V_{0}}{t}$ and transitioning at $2\frac{V_{0}}{t}$, with the root pair locations depending on the $\frac{\Delta V}{\Delta t}$

**FIGURE A2:**
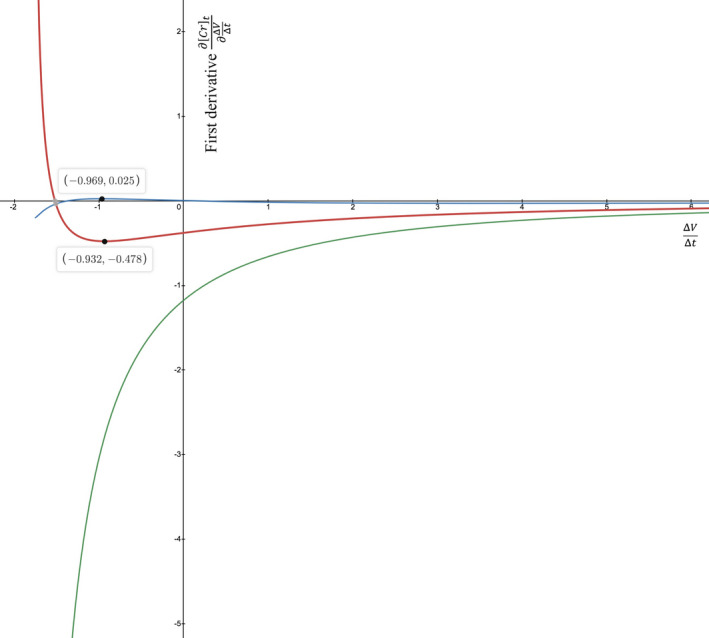
Types of first derivative curves. The $\frac{\partial {\left[Cr\right]}_{t}}{\partial \frac{\Delta V}{\Delta t}}$ (y‐axis) vs. $\frac{\Delta V}{\Delta t}$ (x‐axis) curve takes one of three shapes depending on the ${\mathrm{GFR}}_{K}$. If the ${\mathrm{GFR}}_{K}$ falls into the bottom domain of being $<\frac{\mathrm{Gen}}{{\left[Cr\right]}_{0}}+\frac{V_{0}}{t}$, then the graph has a generic shape like the green curve. It has no minimum or maximum, and it remains below the x‐axis, so all of the $\frac{\partial {\left[Cr\right]}_{t}}{\partial \frac{\Delta V}{\Delta t}}$ values are negative. If the ${\mathrm{GFR}}_{K}$ lies in the middle domain of $\frac{\mathrm{Gen}}{{\left[Cr\right]}_{0}}+\frac{V_{0}}{t}<{\mathrm{GFR}}_{K}<2\frac{V_{0}}{t}$, then the graph has a generic shape like the red curve. It has an absolute minimum, and to the left of the minimum, as $\frac{\Delta V}{\Delta t}\to ‐\frac{V_{0}}{t}$, the $\frac{\partial {\left[Cr\right]}_{t}}{\partial \frac{\Delta V}{\Delta t}}$ values blow up to +∞. To the right of the minimum, the $\frac{\partial {\left[Cr\right]}_{t}}{\partial \frac{\Delta V}{\Delta t}}$ stays negative. If the ${\mathrm{GFR}}_{K}$ is in the top domain of being $>2\frac{V_{0}}{t}$, then the graph has a generic shape like the blue curve. It has an absolute maximum that can be positive, as demonstrated by the blue curve. A small part of the curve is positive, and the rest of the curve is negative in terms of the $\frac{\partial {\left[Cr\right]}_{t}}{\partial \frac{\Delta V}{\Delta t}}$ sign

**FIGURE A3:**
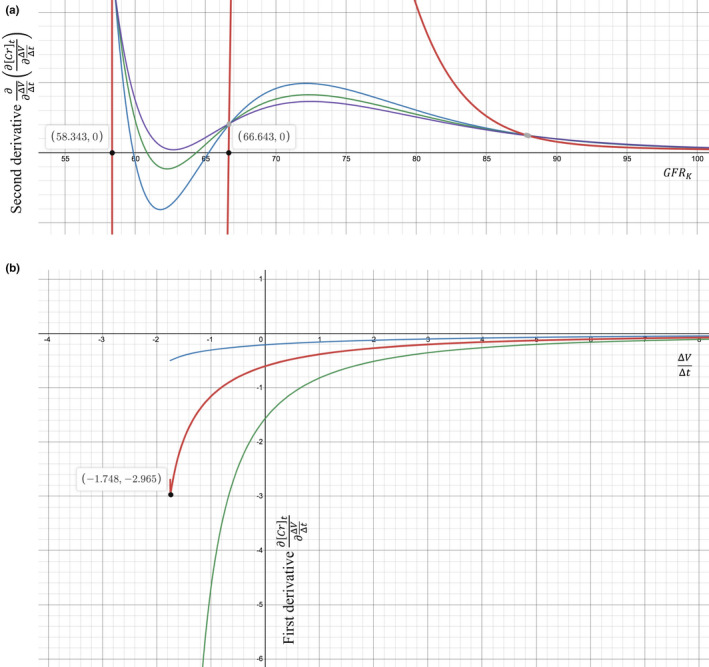
Types of derivative curves when the ${\mathrm{GFR}}_{K}$ roots are reversed. (a) Unlike in Figure A2, the $\frac{\mathrm{Gen}}{{\left[Cr\right]}_{0}}+\frac{V_{0}}{t}$ root may be greater than $2\frac{V_{0}}{t}$, especially when Gen is larger and/or ${\left[Cr\right]}_{0}$ is smaller. The $2\frac{V_{0}}{t}\approx 58.343$ is now the smaller ${\mathrm{GFR}}_{K}$ root of the second derivative, and the $\frac{\mathrm{Gen}}{{\left[Cr\right]}_{0}}+\frac{V_{0}}{t}\approx 66.643$ is the larger root, due to a Gen=150 mg/dL·mL/min and ${\left[Cr\right]}_{0}=4$ mg/dL, while $V_{0}=42$ L, t=24 h, and $\frac{\Delta V}{\Delta t}=‐1.74999$ L/h (red). When $\frac{\Delta V}{\Delta t}$ is barely increased to ‐1.748 (blue), the roots get closer together instead of shifting as a spaced pair to the right, like in Figure A1. Increase $\frac{\Delta V}{\Delta t}$ to ‐1.747 (green) and the roots get closer still, while staying between 58.343 and 66.643. When $\frac{\Delta V}{\Delta t}$ is increased to ‐1.746 (purple), the second derivative no longer has roots. (b) The absence of second derivative roots under most conditions means that the first derivative usually lacks a maximum and a minimum when the ${\mathrm{GFR}}_{K}$ roots are reversed. For ${\mathrm{GFR}}_{K}>66.643$, the typical curve approaches y=0, and the first derivative is always negative (blue). For $66.643>{\mathrm{GFR}}_{K}>58.343$, the curve has an absolute minimum at $\frac{\Delta V}{\Delta t}=‐1.748≅‐\frac{V_{0}}{t}$, and the first derivative is always negative (red). For $58.343>{\mathrm{GFR}}_{K}$, the curve has no maximum or minimum, again, and the first derivative is always negative (green)

## A1.1 ${\mathrm{GFR}}_{K}$ root derivations

How are the 58.34 and 36.67 values derived? They are ${\mathrm{GFR}}_{K}$ roots where the second derivative equals zero as $\frac{\Delta V}{\Delta t}\to ‐\frac{V_{0}}{t}$, since the first derivative maximum and minimum can be successively situated at the leftmost $\frac{\Delta V}{\Delta t}$. If we graph $\frac{\partial }{\partial \frac{\Delta V}{\Delta t}}\left(\frac{\partial {\left[Cr\right]}_{t}}{\partial \frac{\Delta V}{\Delta t}}\right)$ vs. ${\mathrm{GFR}}_{K}$ when $\frac{\Delta V}{\Delta t}$ is nearly leftmost, the two roots lie at about 58.34 and 36.667 (Figure A1, red). The values can be deduced by trying to make $\frac{\partial }{\partial \frac{\Delta V}{\Delta t}}\left(\frac{\partial {\left[Cr\right]}_{t}}{\partial \frac{\Delta V}{\Delta t}}\right)=0$, even as the leftmost $\frac{\Delta V}{\Delta t}$ is setting up Equation (6) to divide by zero in several places. In the graph, $V_{0}=42$, t=24, $\frac{\Delta V}{\Delta t}=‐1.74999$, Gen=60, and ${\left[Cr\right]}_{0}=8$. The larger root, 58.34, comes from setting $1+\frac{{\mathrm{GFR}}_{K}}{\frac{\Delta V}{\Delta t}}$ equal to ‐1, that is, ${\mathrm{GFR}}_{K}=‐2\frac{\Delta V}{\Delta t}$. At the leftmost $\frac{\Delta V}{\Delta t}$, ${\mathrm{GFR}}_{K}=2\frac{V_{0}}{t}$. Reason: If the exponent in ${\left(\frac{V_{0}}{V_{0}+\frac{\Delta V}{\Delta t}t}\right)}^{\left(1+\frac{{\mathrm{GFR}}_{K}}{\frac{\Delta V}{\Delta t}}\right)}$ is ‐1, while the base is a +∞ $\left(\text{due to}\;V_{0}+\frac{\Delta V}{\Delta t}t\to 0^{+}\right)$, the exponential shrinks rapidly to zero. The exponential is multiplied by everything in Equation (6), giving $\frac{\partial }{\partial \frac{\Delta V}{\Delta t}}\left(\frac{\partial {\left[Cr\right]}_{t}}{\partial \frac{\Delta V}{\Delta t}}\right)=0$. Checking the formula, we find that ${\mathrm{GFR}}_{K}=2\frac{V_{0}}{t}=\frac{50}{3}\cdot 2\cdot \frac{42}{24}=58.\bar{3}$. Next, the smaller root, 36.667, comes from setting $\frac{\mathrm{Gen}}{{\mathrm{GFR}}_{K}+\frac{\Delta V}{\Delta t}}‐{\left[Cr\right]}_{0}$ equal to 0, that is, ${\mathrm{GFR}}_{K}=\frac{\mathrm{Gen}}{{\left[Cr\right]}_{0}}‐\frac{\Delta V}{\Delta t}$. At the leftmost $\frac{\Delta V}{\Delta t}$, ${\mathrm{GFR}}_{K}=\frac{\mathrm{Gen}}{{\left[Cr\right]}_{0}}+\frac{V_{0}}{t}$. Around that ${\mathrm{GFR}}_{K}$, Equation (6) is balanced in its tendency to go off to ±∞, giving another $\frac{\partial }{\partial \frac{\Delta V}{\Delta t}}\left(\frac{\partial {\left[Cr\right]}_{t}}{\partial \frac{\Delta V}{\Delta t}}\right)=0$ (Figure A1). Checking the other formula, we find that $\begin{array}{c} {\mathrm{GFR}}_{K}=\frac{\mathrm{Gen}}{{\left[Cr\right]}_{0}}+\frac{V_{0}}{t}=\frac{60}{8}+\frac{50}{3}\cdot \frac{42}{24}=36.\bar{6} \end{array}$.

The two roots encoded by our ${\mathrm{GFR}}_{K}$ formulas are the farthest left that a root pair can go, because they represent the ${\mathrm{GFR}}_{K}$s small enough to shift a first derivative's maximum and then minimum all the way to the leftmost $\frac{\Delta V}{\Delta t}$ (Figure A1, red). For a first derivative's max/min to be located at any $\frac{\Delta V}{\Delta t}$ x‐coordinate to the right of $‐\frac{V_{0}}{t}$, the second derivative's ${\mathrm{GFR}}_{K}$ root pair is going to be larger. To demonstrate, we graphed the second derivative like before but changed the $\frac{\Delta V}{\Delta t}$ from ‐1.74999 to ‐1.7. This plot has less vehement swings, without all of the leftmost $\frac{\Delta V}{\Delta t}$ causing division by zero. As predicted, the ${\mathrm{GFR}}_{K}$ root pair for $\frac{\Delta V}{\Delta t}=‐1.7$ is larger and to the right of the 58.34 and 36.667 pair (Figure A1, blue dotted curve). If we *could* shift the ${\mathrm{GFR}}_{K}$ root pair to the left of $\left(36.67\text{,}\;58.34\right)$, then the smaller root would vanish behind a wall at 36.67, turning the pair into a single. That is why, in a sense, that ${\mathrm{GFR}}_{K}$s between 36.67 and 58.34 lack their partner. That suddenly‐single root relates to the first derivative having only a minimum when $36.67<{\mathrm{GFR}}_{K}<58.34$ (Figure 4c). Further, if we could shift the ${\mathrm{GFR}}_{K}$ root pair to the left of 36.67, then both roots would vanish, forecasting that $\frac{\partial {\left[Cr\right]}_{t}}{\partial \frac{\Delta V}{\Delta t}}$ will lose its minimum next, letting the first derivative plummet toward ‐∞ (Figure 4e).

## A1.2 ${\mathrm{GFR}}_{K}$ domain: curve has a left tail that blows up to be positive

The two ${\mathrm{GFR}}_{K}$ landmarks divide the ${\mathrm{GFR}}_{K}$ number line into three domains. The top domain is ${\mathrm{GFR}}_{K}>2\frac{V_{0}}{t}$, like the ${\mathrm{GFR}}_{K}$ of 100 that was chosen for all of the curves in Figures 2 and 3. ${\mathrm{GFR}}_{K}$s this large allow both the absolute maximum and the relative minimum to be to the right of $\frac{\Delta V}{\Delta t}=‐\frac{V_{0}}{t}$ (Figure A2, blue). If ${\mathrm{GFR}}_{K}$ descends into the middle domain of $2\frac{V_{0}}{t}>{\mathrm{GFR}}_{K}>\frac{\mathrm{Gen}}{{\left[Cr\right]}_{0}}+\frac{V_{0}}{t}$, then the $\frac{\partial {\left[Cr\right]}_{t}}{\partial \frac{\Delta V}{\Delta t}}$ curve has shifted far enough left to still have a minimum but also to vanish/transition the absolute maximum into the left tail of a curve that blows up to +∞. We graphed an example of this curve shape using ${\mathrm{GFR}}_{K}=52$ ml/min, which is between 58.34 and 36.67 (Figure A2, red).

In special cases, the left tail may remain negative. If the two ${\mathrm{GFR}}_{K}$ roots are so close that the middle domain is squeezed, then an absolute minimum is forced to be near the leftmost $\frac{\Delta V}{\Delta t}$, a wall that truncates the left tail before $\frac{\partial {\left[Cr\right]}_{t}}{\partial \frac{\Delta V}{\Delta t}}$ can increase to a positive value, much less blow up to +∞.

## A1.3 ${\mathrm{GFR}}_{K}$ domain: curve has no maximum or minimum and stays negative

If ${\mathrm{GFR}}_{K}$ lies in the bottom domain of $\frac{\mathrm{Gen}}{{\left[Cr\right]}_{0}}+\frac{V_{0}}{t}>{\mathrm{GFR}}_{K}$, then the $\frac{\partial {\left[Cr\right]}_{t}}{\partial \frac{\Delta V}{\Delta t}}$ curve has shifted so far to the left that even the minimum has been transitioned into the left tail of a curve that plunges toward ‐∞ (Figure A2, green). To the right, the curve asymptotically approaches y=0 from the negative side. The absence of both a maximum and a minimum is corroborated by the fact that the second derivative cannot equal zero when the ${\mathrm{GFR}}_{K}<\frac{\mathrm{Gen}}{{\left[Cr\right]}_{0}}+\frac{V_{0}}{t}$ (Figure A1). We graphed an example of this curve shape using ${\mathrm{GFR}}_{K}=32$ ml/min, which is below 36.67 (Figure A2, green).

## A1.4 ${\mathrm{GFR}}_{K}$ root reversal keeps $\frac{\partial {\left[Cr\right]}_{t}}{\partial \frac{\Delta V}{\Delta t}}$ negative

The second derivative as $\frac{\Delta V}{\Delta t}\to ‐\frac{V_{0}}{t}$ has two ${\mathrm{GFR}}_{K}$ roots, but $\frac{\mathrm{Gen}}{{\left[Cr\right]}_{0}}+\frac{V_{0}}{t}$ does not have to be $<2\frac{V_{0}}{t}$. What if the order of the roots is reversed, because Gen is larger and/or ${\left[Cr\right]}_{0}$ is smaller? Then the second derivative behaves differently. Its ${\mathrm{GFR}}_{K}$ root pair exists only transiently. Before, the root pair of $\left(\frac{\mathrm{Gen}}{{\left[Cr\right]}_{0}}+\frac{V_{0}}{t},2\frac{V_{0}}{t}\right)$ was the smallest since it was at the leftmost $\frac{\Delta V}{\Delta t}$, so root pairs at any $\frac{\Delta V}{\Delta t}>‐\frac{V_{0}}{t}$ would get larger, but at least the pairs would persist (Figure A1). Now, when the roots are reversed, the pairs no longer shift to the right. They stay confined between $2\frac{V_{0}}{t}$ and $\frac{\mathrm{Gen}}{{\left[Cr\right]}_{0}}+\frac{V_{0}}{t}$, and the two roots get *closer* to each other as $\frac{\Delta V}{\Delta t}$ is increased. Even a minuscule increase from $‐\frac{V_{0}}{t}$ is sufficient to close the gap between the two roots. After that, the second derivative lies above the x‐axis and does not have any roots (Figure A3a, purple). If the root pairs cease to exist, then the concept of ${\mathrm{GFR}}_{K}$ domains becomes mostly irrelevant. That said, the middle domain does contain all of the roots.

With roots reversed, the first derivative almost always lacks a maximum and a minimum. The stereotypical shape of this $\frac{\partial {\left[Cr\right]}_{t}}{\partial \frac{\Delta V}{\Delta t}}$ curve is to increase from a left endpoint at $\frac{\Delta V}{\Delta t}=‐\frac{V_{0}}{t}$ toward y=0, asymptotically from the negative side (Figure A3b, blue or green). The window for having two ${\mathrm{GFR}}_{K}$ roots of the second derivative is narrowly open when $\frac{\Delta V}{\Delta t}$ is in the vicinity of $‐\frac{V_{0}}{t}$. If $\frac{\Delta V}{\Delta t}≅‐\frac{V_{0}}{t}$ and the ${\mathrm{GFR}}_{K}$ lies between the smaller $2\frac{V_{0}}{t}$ and the larger $\frac{\mathrm{Gen}}{{\left[Cr\right]}_{0}}+\frac{V_{0}}{t}$, flipped due to root reversal, then the $\frac{\partial {\left[Cr\right]}_{t}}{\partial \frac{\Delta V}{\Delta t}}$ curve has an absolute minimum (all roots in this ${\mathrm{GFR}}_{K}$ domain are minimums), and the left tail gets truncated by the $‐\frac{V_{0}}{t}$ wall before the first derivative can turn positive (Figure A3b, red). Overall, the combination of larger Gen and smaller ${\left[Cr\right]}_{0}$ is not conducive to a first derivative being positive. A necessary but not sufficient condition for first derivative positivity, judging by root order, is that $\frac{\mathrm{Gen}}{{\left[Cr\right]}_{0}}+\frac{V_{0}}{t}$ should at least be $<2\frac{V_{0}}{t}$, or $\frac{\mathrm{Gen}}{{\left[Cr\right]}_{0}}<\frac{V_{0}}{t}$. Further restrictions on $\frac{\mathrm{Gen}}{{\left[Cr\right]}_{0}}$ apply, in order for the first derivative to be positive (see Sections 3.4 and 3.5).
